# Amyloidosis in Retinal Neurodegenerative Diseases

**DOI:** 10.3389/fneur.2016.00127

**Published:** 2016-08-08

**Authors:** Ambra Masuzzo, Virginie Dinet, Chelsea Cavanagh, Frederic Mascarelli, Slavica Krantic

**Affiliations:** ^1^Centre de Recherche des Cordeliers, Institut national de la santé et de la recherche médicale (INSERM), Université Paris Descartes, Sorbonne Paris Cité, UMR_S 1138, Université Pierre et Marie Curie Université Paris 06, Sorbonne Universités, Paris, France; ^2^Department of Neuroscience, Douglas Hospital Research Center, Montreal, QC, Canada

**Keywords:** Alzheimer’s disease, age-related macular degeneration, glaucoma, neurodegeneration, synaptic and mitochondrial dysfunction, micoangiopathy, neuroinflammation

## Abstract

As a part of the central nervous system, the retina may reflect both physiological processes and abnormalities related to pathologies that affect the brain. Amyloidosis due to the accumulation of amyloid-beta (Aβ) was initially regarded as a specific and exclusive characteristic of neurodegenerative alterations seen in the brain of Alzheimer’s disease (AD) patients. More recently, it was discovered that amyloidosis-related alterations, similar to those seen in the brain of Alzheimer’s patients, also occur in the retina. Remarkably, these alterations were identified not only in primary retinal pathologies, such as age-related macular degeneration (AMD) and glaucoma, but also in the retinas of Alzheimer’s patients. In this review, we first briefly discuss the biogenesis of Aβ, a peptide involved in amyloidosis. We then discuss some pathological aspects (synaptic dysfunction, mitochondrial failure, glial activation, and vascular abnormalities) related to the neurotoxic effects of Aβ. We finally highlight common features shared by AD, AMD, and glaucoma in the context of Aβ amyloidosis and further discuss why the retina, due to the transparency of the eye, can be considered as a “window” to the brain.

## Introduction

Pathological alterations, such as synaptic dysfunctions, neuronal cell loss, inflammatory responses, microvasculature abnormalities, mitochondrial failure, and oxidative stress, have been associated with amyloid-beta (Aβ) in the brain. However, similar pathological alterations have more recently also been reported in the retina where they may mirror analogous events occurring in the brain (1). The present review will focus on these aforementioned aspects of Aβ’s deleterious effects but does not have the ambition to cover all aspects of Aβ cytotoxicity. For instance, the issues related to aberrant Aβ clearance will not be discussed here since they have been recently extensively reviewed elsewhere [e.g., Ref. (2)].

Retinal accumulation of Aβ is broadly recognized as being involved in amyloidosis-associated neurodegeneration. Pathological hallmarks of amyloidosis are related to the accumulation of specific types of proteins, including Aβ, prone to oligomerize with a high content of beta (β)-sheet structures (3). Among the neurodegenerative diseases related to Aβ amyloidosis, Alzheimer’s disease (AD) is certainly the best known and the most studied. More recently, it has been recognized that Aβ-related amyloidosis also occurs during glaucoma and age-related macular degeneration (AMD). Historically and up to very recently, AD was considered as an exclusively cerebral disorder, while glaucoma and AMD were regarded as neurodegenerative disorders specific to the retina. However, it is increasingly clear that AD-like pathological alterations seen in the brain also occur in the retina (4), where they may even start earlier. Conversely, the pathological phenomena observed in glaucoma, for example, are associated with neurodegeneration of selected brain areas (5). Altogether, this new evidence suggests that the retina may be used as the “window” to the brain for the study of the earliest pathophysiological changes involved in neurodegeneration. This attractive idea is behind different aspects of amyloidosis that will be discussed here.

Parkinson’s disease (PD), which shares many features of Aβ-amyloidosis with AD, glaucoma, and AMD, will not be discussed here, and we recommend a number of excellent and exhaustive reviews on this topic (6, 7). Indeed, although PD is considered an amyloidosis-associated disease, involving the accumulation of both Aβ and α-synuclein, the relevant fibrils have not been identified in the PD retina (8). This is in sharp contrast with the presence of Aβ plaques, identical to those found in AD-vulnerable brain areas that have been identified in the retina (9, 10). Furthermore, Aβ-amyloidosis seen in PD is sometimes considered as an epiphenomenon to the oligomerization of α-synuclein into structures known as Lewy bodies. Consequently, rigorous analysis of alterations specific to Aβ-amyloidosis in PD would require a systematic comparative follow-up of cohorts composed of “mixed” PD (displaying both α-synuclein and Aβ-amyloidosis) and “pure” PD (displaying exclusively α-synuclein amyloidosis). Such studies, similar to the one reported by Bertrand and colleagues (11), are still relatively scarce. Finally, there is no consensus about the precise type of pathological alterations in the PD retina, since thickening (12), thinning (13), and absence of change (14) in the retinal nerve fiber layer (RNFL) have all been reported. The analysis of retinal Aβ-amyloidosis in PD would therefore be more complicated. By consequence, this review will focus only on AD, glaucoma, and AMD.

## Biology of Amyloid-β and Its Precursor APP

Amyloid precursor protein (APP), a type 1 transmembrane glycoprotein, belongs to a family of proteins, which in mammals include APP-like protein-1 (APLP1) and APP-like protein-2 (APLP2) (15). Despite the widespread expression of the APP gene in mammalian and non-mammalian cells, the physiological role of APP is still unclear. APP-related mRNA has been found not only in the nervous system but also in the immune system, muscles, and other organs, such as the pancreas, lung, and kidney (16, 17). Alternative splicing of APP mRNA gives rise to multiple isoforms, which are differentially expressed among various tissues and different stages of development. In particular, APP is upregulated during brain development, and specific APP variants are associated with neurite outgrowth and synaptogenesis (18, 19).

**Figure 1:**
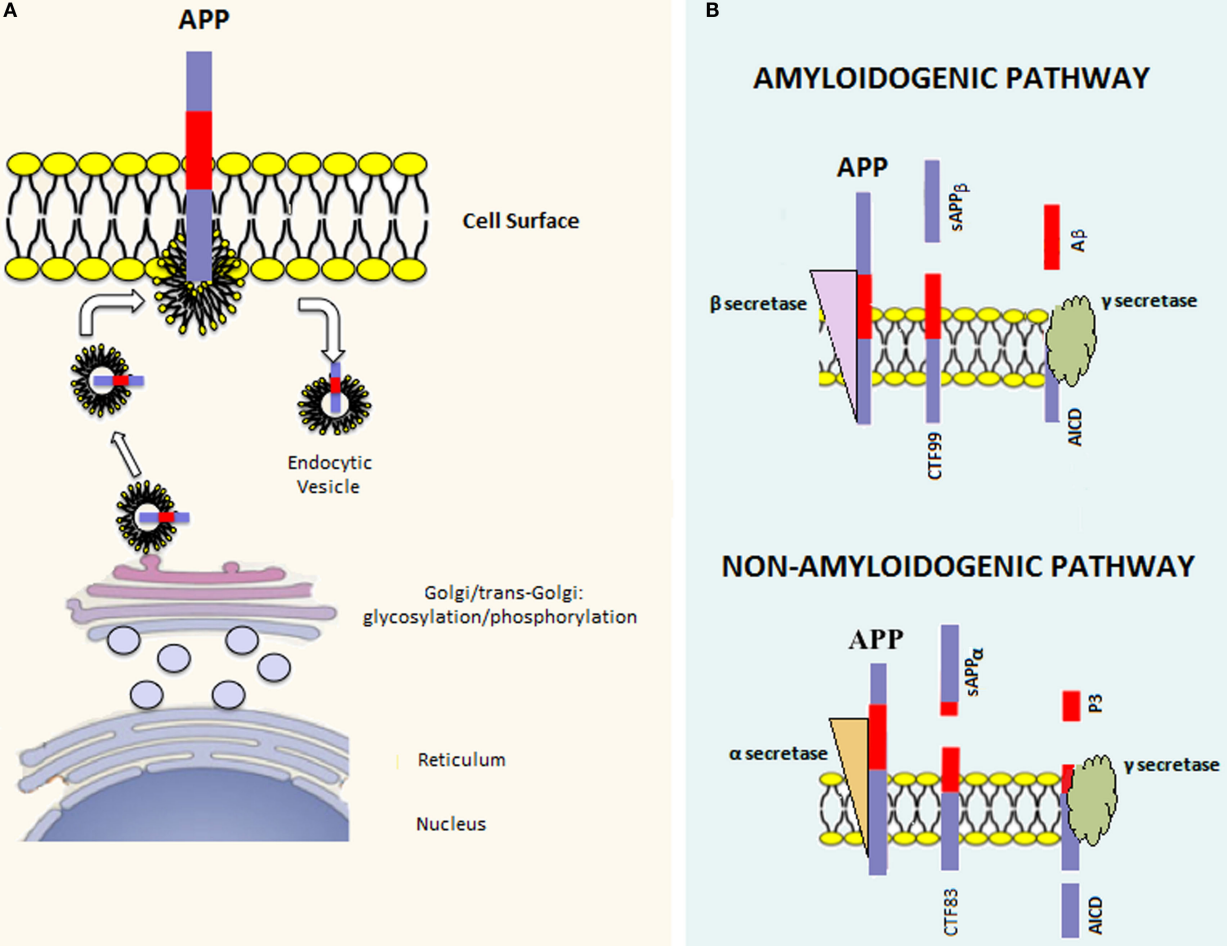
**APP processing**. **(A)** Once the APP mRNA is translated, the nascent polypeptide undergoes posttranslation modifications (e.g., glycosylation and phosphorylation) in the endoplasmic reticulum and Golgi apparatus. Afterward, the mature APP protein is addressed to the cell surface through the constitutive secretion pathway. At this point, APP is internalized in endocytic vesicles. Endosomal APP can be recycled to the cell surface or degraded through the lysosomal system. **(B)** APP can be processed through two distinct pathways. The amyloidogenic pathway involves cleavage by β-secretase, which leads to the formation of the carboxy-terminal fragment 99 (CTF99) and soluble APPβ (sAPPβ). This cleavage is followed by a second cleavage mediated by γ-secretase that leads to the formation of the APP intracellular domain (AICD) and Amyloid-β (Aβ). On the other hand, the non-amyloidogenic pathway involves the sequential cleavage first by α-secretase, which leads to the formation of the carboxy-terminal fragment 83 (CTF83) and soluble APPα (sAPPα), followed by γ-secretase cleavage, which leads to the formation of AICD and P3.

There are three major APP isoforms, APP770, APP751, and APP695, which are all generated from the alternative splicing of exons 7 and/or 8. APP695 is mainly neuronal, whereas the other two variants are principally non-neuronal (20). APP polypeptides undergo posttranslational modifications (such as glycosylation and phosphorylation) and are subsequently addressed to the plasma membrane *via* the constitutive secretory pathway (Figure 1A). Successively, APP is internalized through clathrin-mediated endocytosis and reaches the endosomal system. Part of endosomal APP is recycled to the cell surface, whereas another conspicuous part is degraded in lysosomes (21, 22). In the steady state, APP is preferentially localized in the Golgi and in the trans-Golgi network, and only a tiny fraction is localized on the cell surface.

Amyloid precursor protein can be posttranslationally processed through two distinct pathways [reviewed in Ref. (23)] (Figure 1B). The amyloidogenic pathway involves sequential cleavage steps by β-secretase and γ-secretase, which generates Aβ. The second pathway, which is predominant, involves sequential cleavage steps by α-secretase and γ-secretase but does not yield Aβ. Indeed, α-secretase cleavage occurs within the Aβ peptide region, preventing Aβ formation. It has been shown that proteases belonging to the A-disintegrin and metalloproteinase (ADAM) family have α-secretase activity (24–26). Since ADAMs are cell surface proteins, α-secretase cleavage likely occurs at the level of the plasma membrane and involves the membrane pool of APP (27). α-secretase cleavage leads to the formation of an amino-terminal fragment called secreted APP (sAPP)α and a carboxy-terminal fragment called CTF83. β-secretase is a type 1 transmembrane protease, and its cleavage leads to the formation of sAPPβ and CTF99. In converse to α-secretase cleavage, β-secretase cleavage occurs mainly in endocytic vesicles and not at the cell surface, where both β-site APP cleaving enzyme-1 (BACE1) and APP are swiftly recycled. The first cleavage step is followed by γ-secretase cleavage in both pathways. The latter is a protein complex composed of at least four proteins: presenilin (PS) 1 or 2, nicastrin, presenilin enhancer 2 (Pen 2), and anterior pharynx defective 1 (Aph-1) (28). γ-secretase processes CTF83 and CTF99, yielding the APP intracellular domain (AICD) plus the fragment p3 and AICD plus Aβ, respectively. Aβ peptides of different lengths, from 38 to 43 amino acids, can be generated by γ-secretase cleavage; however, Aβ1–42 and Aβ1–40 are considered to be the most relevant forms to amyloidosis.

Since APP undergoes sequential cleavage steps, it has been difficult to distinguish the physiological role of APP from those of its cleavage products. Generally, the role of APP in the brain is regarded as beneficial and often associated with its cleavage product, sAPPα. It has been shown that APP promotes cell proliferation, neuronal stem cell differentiation, neurite outgrowth, synaptogenesis, cell adhesion, and regulates long-term potentiation (LTP). APP-KO mice are viable and fertile, suggesting that APP – or its products – are not essential for development or alternatively, are part of a network of proteins with redundant functions (29). However, APP-deficient mice present various abnormalities, such as reduced body and brain size, hypersensitivity to seizures, and impaired learning and LTP. These phenotypes are rescued by the introduction of sAPPα in APP-deficient mice, suggesting that sAPPα may play an important role in brain development and function (30).

Compared with sAPPα, little is known about the putative physiological roles of other cleavage products from the non-amyloidogenic and amyloidogenic pathways. However, it has been proposed that Aβ may regulate synaptic activity, although controversial results have been reported on its beneficial versus deleterious effects (31, 32). In addition, Aβ may be involved in the control of cholesterol transport (33) and lipid homeostasis (34). For instance, direct activation of sphingomyelinase and inhibition of hydroxymethylglutaryl-CoA reductase (HMGR) by Aβ1–42 and Aβ1–40 have been demonstrated (35). The question of the physiological role of Aβ remains open, and further studies are clearly needed in this field.

## Amyloid-β and Its Precursor in the Eye and Retina

The retina is a highly specialized neurosensory tissue, which lines the back of the eye. It is an integral part of the brain comprising six different types of neuronal cells and two types of macroglia cells: retinal Müller glial cells and astrocytes. Retinal and central nervous system (CNS) neurons are derived from common progenitors (36). Differentiated retinal neurons are organized into a well-defined laminar structure and are distributed into three cell and two synaptic layers. The outer segment of the retina is populated by two different types of photoreceptors: cones and rods, which are able to detect light and form the outer nuclear layer (ONL). The detected light signal is transmitted to the cells located at the inner nuclear layer (INL), mainly the bipolar cells followed by retinal ganglion cells (RGCs), either directly or indirectly *via* type II amacrine cells. The latter, together with horizontal cells, modulate glutamatergic neurotransmission along the main synaptic axis comprising photoreceptors, bipolar, and ganglion cells. The principal function of the INL cells is to integrate and regulate the signal input. The RGC axons converge into the optic nerve fibers, which convey the signal to the visual cortex (37).

To date, the physiological roles of APP in the retina have not been extensively investigated, although a consensus has been met about its expression by retinal pigmented epithelial (RPE) cells in the healthy retina (38). The role of APP in the development of the mouse retina has been recognized such that APP is required for the full differentiation of the AII subtype of amacrine cells. Similar to its role in the brain, APP may be implicated in retinal synaptogenesis. Indeed, APP participates in the developmental determination of the inner plexiform layer (IPL), where amacrine cells synapse to bipolar and ganglion cells (39). Concerning the physiological role of APP in adult mice, it has been shown that APP regulates inner retinal layer function. Indeed, APP-KO mice display alterations in the rod and cone pathways. However, these mice do not present any major deficits in visual function; therefore, APP is not likely a required factor (40). Among all retinal neurons, at least in the rabbit, ganglion cells are the sole cells able to synthesize and express APP on their plasma membrane in the absence of any pathological insult (41). In the human retina, APP expression is age-dependent and was revealed in RGC neurons and the RNFL (42).

Concerning Aβ, there is no published data on its putative physiological role in the retina. Of interest, the expression of BACE1 has been recently reported in the blood–brain barrier endothelial cells of mouse, bovine, and human origin, thus suggesting putative local production of Aβ in cerebral blood vessels (43). It remains unknown whether retinal vessel endothelial cells display analogous BACE1 expression. By contrast, BACE1 expression has been reported in the plexiform layer of the rat retina pointing to its synaptic localization (44).

The other parts of the eye have been much less studied in terms of the expression and function of APP and its cleavage products. However, both APP and the proteolytic enzymes involved in its cleavage were found to be expressed in some other eye compartments. For instance, APP and the secretases involved in its processing were identified in the lens (45). Similarly, Aβ was identified both in the lens (46) and in the vitreous fluid (47).

## Pathological Accumulation of Amyloid-β: Amyloidosis, Amyloidopathy, and Amyloidogenesis

Different terms have been associated with the pathological accumulation of Aβ, with amyloidosis historically being used first. Amyloidosis is a broad term designating a metabolic disease characterized by the extracellular accumulation of globular or natively unfolded or misfolded amyloidogenic polypeptides. Amyloidogenic polypeptides contain a high proportion of β-sheets and have a great propensity to aggregate into highly organized and kinetically stable amyloid fibrils, amorphous aggregates, or oligomers. To date, more than 20 precursor proteins of fibrils (including APP) have been identified in systemic and localized amyloidosis (3). A remarkable property of these fibrils is that, independent of the type of the precursor protein, they are all 80–100Å in width. Furthermore, these fibrils organize in a tridimensional β-pleated sheet conformation with the direction of the polypeptide backbone perpendicular to the fibril axis (cross-beta structure). Another remarkable characteristic of amyloidogenic peptides and derived aggregates is their affinity for the Congo red stain (48). The Aβ-related amyloidopathies consist of increased intra- and/or extracellular accumulation of Aβ and deposition of Aβ in the form of insoluble material, such as amyloid plaques or drusens. Several disorders are associated with amyloidopathies, and most of them are neurodegenerative diseases (e.g., AD, PD, polyglutamine diseases, prion disorders, and AMD).

Amyloid-beta is produced *via* the amyloidogenic pathway of APP processing. However, the mechanisms by which this pathway may take over the non-amyloidogenic pathway are poorly understood, especially considering that both pathways coexist in physiological conditions (49). Many genetic and epigenetic factors may be involved, but the evidence points to an increase in the ratio of β- over α-secretase activity as a trigger. This change in the subtle balance between secretase activities in physiological conditions might be associated with the positive control of β-secretase activity by its substrate APP and directly related to APP overexpression and subsequent increase in Aβ production (50). Over the course of normal aging, Aβ is deposited subretinally in the mouse and human retina (51). With age, Aβ accumulates at the interface of the RPE and the photoreceptor outer segment tips. This finding is consistent with increased Aβ1–42 secretion by aged human RPE cells (52). As Aβ accumulates subretinally, microglial cells in normal aged mice become bloated with cellular debris and Aβ (51). The accumulation of Aβ in the subretinal space might contribute to the 23–30% reduction in photoreceptors that occurs over human lifetimes (53).

## Amyloid-β Aggregation and Toxicity

An increase in Aβ production above normal physiological levels yields cytotoxicity. Among most common Aβ species (i.e., 1–40 and 1–42 amino acid-containing isoforms), Aβ1–42 is considered the most neurotoxic as it is more prone to oligomerization (54). The amyloid aggregation pathway is still poorly understood and several intermediates are likely involved. Small soluble Aβ monomers can interact to form Aβ oligomers in the extracellular space. Aβ oligomers aggregate to form larger fibrils, which in turn aggregate to form extracellular plaques. The mechanisms of Aβ toxicity are still unclear, and different hypotheses have been proposed. According to the original “amyloid-β cascade hypothesis,” insoluble amyloid fibrils are the main molecular culprit underlying toxicity (55). More recently, this hypothesis has been revised to the “oligomeric amyloid-β hypothesis” (56). It is currently believed that the most toxic intermediates are small oligomers (with degree of polymerization lower than 10), also known as amyloid-β diffusible ligands (ADDL) or protofibrils. The latter have a bigger diffusivity and a larger surface-to-volume ratio that leads to the exposure of hydrophobic patches (57). However, it is not yet clear which oligomeric species is “the most” toxic since dimers/trimers (58), tetramers (59), and duodecamers such as Aβ*56 (60) have all been considered as plausible candidates depending on the paradigm (*in vivo, in vitro*) or species (murine, human) studied.

Soluble Aβ oligomers, although they are certainly not involved in all the aspects of AD, are still regarded as key initial triggers of pathogenesis (61). The bioactive pool of soluble Aβ comprises two fractions: the first is generated in the endosomal compartment and secreted into the extracellular space by exocytosis and the second is intracellular and has been found in both AD patients and animal models of the disease (62, 63). Cellular mechanisms by which soluble oligomers exert neurotoxic effects are multifaceted, involving synaptotoxicity and mitochondrial dysfunction likely related to oxidative stress and metabolic impairment. Insoluble Aβ aggregates also contribute to Aβ toxicity either directly through the release of soluble oligomers (64) or indirectly *via* adaptive cellular responses, such as glial and endothelial activation, which can yield neuroinflammation (65) and Aβ-related angiopathy (66), respectively.

### Amyloid-β and Synaptic Dysfunction

**Figure 2:**
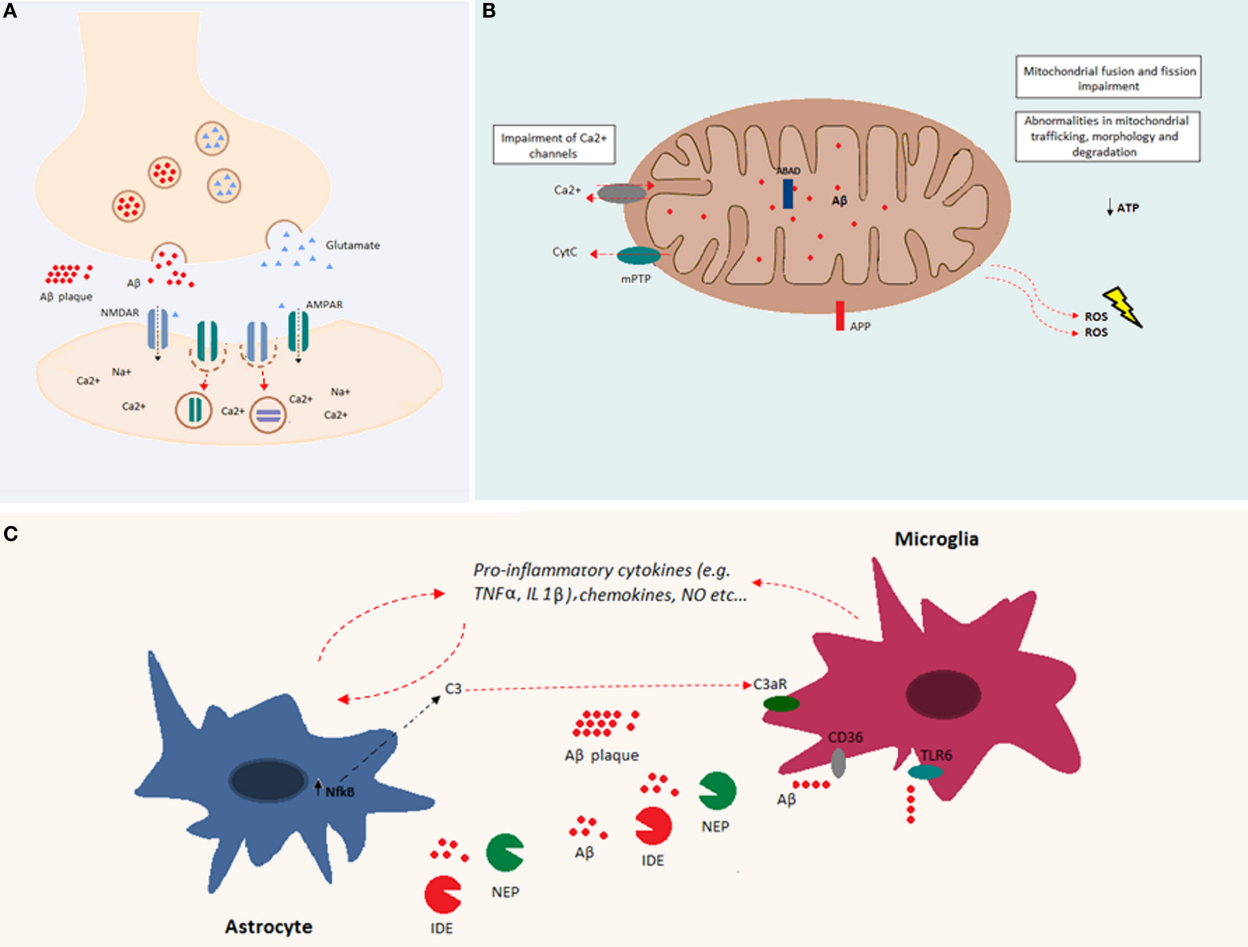
**Overview of Aβ cellular effects**. **(A)** Aβ is implicated in synapse loss. Increased Aβ at the synapse reduces excitatory postsynaptic transmission. Indeed, Aβ oligomers reduce glutamatergic synaptic transmission by decreasing the number of both AMPA and NMDA receptors at the postsynaptic membrane. **(B)** Aβ accumulation within the mitochondria causes impairments in fusion and fission and abnormalities in mitochondrial trafficking, morphology, and degradation. Both APP and Aβ can interact with mitochondrial membranes. Aβ, by interacting with mitochondrial respiratory enzymes, causes decreased ATP production and increased reactive oxygen species (ROS) production. In addition, Aβ binds the Aβ-binding alcohol dehydrogenase (ABAD), increasing its deleterious effects in mitochondrial function. Mitochondrial Ca^2+^ channels are impaired by Aβ, and mitochondrial permeability transition pore (mPTP) opening gives rise to the enhancement of cytochrome *c* (Cyt*c*) release. **(C)** Aβ accumulation induces glial activation. Astrocytes and microglia release cytokines, chemokines, and nitric oxide (NO) after exposure to Aβ. Increased levels of NFκB in astrocytes induce the release of C3, which binds the C3a receptor, impairing microglia-mediated Aβ phagocytosis. Both microglia and astrocytes release Aβ-degrading proteases, such as neprilysin and insulin-degrading enzyme. Aβ fibrils are degraded through microglia-dependent phagocytosis, triggered by the ligation of Aβ to microglia receptors (e.g., CD36 and TLR-6).

One of the prominent facets of Aβ toxicity concerns synaptic loss (67). This toxicity may be related to a deviation from the Aβ-associated modulation of synaptic excitability under physiological conditions (31). Indeed, increased synaptic activity may enhance Aβ release at the synaptic level, reducing excitatory postsynaptic transmission. In particular, it has been shown both *in vitro* and *in vivo*, that Aβ oligomers reduce glutamatergic synaptic transmission by decreasing the number of α-amino-3-hydroxy-5-methyl-4-isoxazolepropionic acid (AMPA) and *N*-methyl d-aspartate (NMDA) receptors at the synapse (68–71) (Figure 2A). A decrease in AMPA receptors by Aβ has been related to increased phosphorylation of the Ca^2+^-permeable subunit, GluR2, and a subsequent increase of intracellular Ca^2+^ levels (72). A decrease in NMDA receptors by Aβ involves a similar mechanism *via* dephosphorylation of the NR2B subunit and subsequent increase in receptor endocytosis (73). Thus, Aβ is part of a refined regulatory circuit in which intermediate levels of Aβ are correlated with a physiological increase in presynaptic activity, whereas lower or higher Aβ levels are correlated with reduced presynaptic and postsynaptic transmission, respectively (74). Likely, Aβ differentially affects synaptic activity, depending on synapse type, neuron type, and/or brain region, leading to the imbalance and instability of neuronal networks (75). At the cellular level, Aβ-mediated alterations involve a shift toward increased excitability manifesting in a decreased resting potential of the neuronal membrane (76). Similarly, the addition of exogenous Aβ oligomers to hippocampal neurons induced hyperpolarization of the action potential (AP) threshold and decreased after-hyperpolarization (AHP), both compatible with an increase in neuronal excitability (77).

Of note, the vast majority of the above-discussed data has been obtained *in vitro*, by treating cerebral (hippocampal or cortical primary) neurons with soluble Aβ oligomers. Analogous data for retinal neurons are scarce, although it has been reported that intravitreal injection of Aβ triggers acute photoreceptor cell death and delayed RGC apoptosis (78). The latter likely involves an indirect mechanism *via* the activation of Müller cells (78). Finally, a similar Aβ challenge by intravitreal injection resulted in an impaired pattern of acetylcholine, γ-aminobutyric acid (GABA), and serotonin neurotransmitter expression with catecholaminergic markers being relatively unaffected (79).

### Amyloid-β and Mitochondrial Dysfunction

Mitochondrial dysfunction is a common feature of various neurodegenerative diseases and causes alterations in mitochondrial respiratory enzyme complex activities, oxidative stress, opening of mitochondrial permeability transition pores (mPTPs), and enhanced apoptosis (80). In the brain, intracellular Aβ has been associated with axonopathy and apoptosis initiation (81, 82). Moreover, in neurons, mitochondrial dysfunction is also associated with increased susceptibility to excitotoxicity (i.e., cell death caused by excessive stimulation of neurons by excitatory amino acids, such as glutamate) (83).

Soluble Aβ peptides have been found in different organelles, and their deleterious effects are largely due to their accumulation within mitochondria. Indeed, intracellular Aβ inhibits the activity of different mitochondrial respiratory enzymes, causes decreased ATP production, and increases the production of reactive oxygen species (ROS) (84–87) (Figure 2B). Moreover, Aβ induces mitochondrial dysfunction by interacting with the Aβ-binding protein known as Aβ-binding alcohol dehydrogenase (ABAD), which is present on the mitochondrial membrane (88). In addition, Aβ accumulation impairs the permeability of mitochondrial membranes leading to the opening of mitocholdrial Ca^2+^ channels and mPTPs as well as the enhancement of cytochrome *c* (Cyt*c*) release (89). At the structural level, accumulation of soluble Aβ impairs mitochondrial fusion and fission and triggers abnormalities in mitochondrial trafficking, morphology, and degradation [reviewed in Ref. (90)].

In the retina, intraocular injection of respiratory complex (I, III, and IV) inhibitors or Aβ fibrils yields induction of BACE1 expression and activity, suggesting that Aβ-mediated mitochondrial respiratory inhibition and oxidative stress facilitate BACE1 expression (44). Interestingly, subretinal injection of Aβ oligomers resulted in RPE cell hypertrophy without triggering apoptosis but yielded a significant amount of delayed photoreceptor death (91).

### Amyloid-β and Glial Activation

The presence of misfolded proteins and their aggregates causes an alteration in the receptor–ligand interactions that modulate both microglia and astroglia activity. Both microglia and astroglia release cytokines, nitric oxide, and other cytotoxic molecules after Aβ exposure (Figure 2C). Astroglia regulate synapse formation and function in addition to participating in the tripartite synapse (92). It was shown that Aβ upregulates NFκB in astrocytes, leading to C3 release (93). The latter binds the neuronal G-protein-coupled receptor C3aR, inducing dendritic structural alterations and synaptic dysfunction. C3 also interacts with microglial C3aR causing alterations in cognitive function and impairment of Aβ phagocytosis (94). Moreover, the exposure of astroglia to Aβ, favors astrogliosis, a process that leads to molecular and functional changes in astrocytes and is implicated in different brain diseases (95). Furthermore, astroglia play an important role in Aβ clearance. Indeed, astrocytes are able to bind to and degrade Aβ and release extracellular Aβ-degrading proteases (e.g., neprilysin, insulin-degrading enzyme, angiotensin-converting enzyme-1, and endothelin-converting enzyme-2) (96, 97). On the other hand, microglia are phagocytic cells ubiquitously distributed in the brain. Microglia play important roles in the maintenance and plasticity of neuronal circuits, in the surveillance for pathogens or cell debris, and in tissue maintenance (98–100). Aβ oligomers and fibrils are able to bind microglia surface receptors, such as cluster of differentiation-36 (CD36), toll-like receptor (TLR)-4, and TLR-6, leading to their activation (101, 102). Activated microglia release proinflammatory cytokines and chemokines (102, 103). Consequently, extracellular proteases (in particular, neprylysin and the insulin-degrading enzyme) are released and give rise to enzymatic degradation of soluble Aβ (104). In addition, receptor ligation triggers the activation of microglial-dependent phagocytosis of Aβ fibrils and their degradation through the endolysosomal pathway. Aβ accumulation itself leads to increased release of proinflammatory cytokines, such as tumor necrosis factor-alpha (TNFα), interleukin (IL)-1α, and IL-1β (105, 106). The massive release of proinflammatory cytokines might be associated with impairment of synaptic transmission by suppressing LTP (100). It has also been shown that there is a positive feedback loop between TNFα and Aβ, since TNFα is able to induce Aβ production by increasing BACE1 expression and γ-secretase activity (107, 108). In addition, the use of TNFα inhibitors leads to a decrease in APP processing and Aβ (109). Similarly, IL-1β increases Aβ production by increasing γ-secretase activity (107). Even though the early activation of astroglia and microglia is beneficial and leads to Aβ clearance, in a pathological context, the sustained activation of these cells may induce a positive feedback loop between APP processing and inflammation, which is deleterious (100). Indeed, inflammation is a consequence of Aβ accumulation, and as a result, inflammation contributes directly to the pathogenesis and progression of the disease.

### Amyloid-β and Blood Vessels

The pathophysiological cause and consequence of the accumulation of Aβ and/or its precursor APP in the brain and the retina remain poorly understood. Twenty years ago, it was reported that coincident APP and B-cell lymphoma-2 (Bcl-2) induction may play a role in rat retinal cell survival after optic nerve and vascular injury. The underlying mechanism involves APP induction selectively in either activated astrocytes (Müller cells) or neurons (110). Microinjection of Aβ into the adult zebrafish eye triggers an increase in endothelial tip cells and a subsequent increase in the capillary bed density without affecting larger arterial vessels (111). In this light, the recent discovery of BACE1 expression in endothelial cells (indicating local cleavage of APP to Aβ in the blood–brain barrier in mice, bovine, and humans) has attracted much interest (43). Indeed, BACE1 appears to be a critical regulator of retinal homeostasis since genetic invalidation of BACE1 in mice yields retinal thinning, apoptosis, reduced retinal vascular density, and increased accumulation of the age pigment, lipofuscin (112). The use of BACE1 inhibitors for therapeutic purposes should therefore be carefully evaluated for the putative impairment of retinal homeostasis.

Some aspects of endothelial BACE1 regulation have been elucidated, such as its induction in the presence of reduced levels of microRNA-195 (miR-195) in hypoperfusion/hypoxic conditions (113). This BACE1 induction is associated with reduced occludin expression in tight junctions of cerebral blood vessels (114). The cellular mechanism behind the deleterious effects of Aβ on cerebral vessel endothelial cells involves activation of the cationic Ca^2+^-permeable channel transient receptor potential melastatin-2 (TRPM-2) and intracellular Ca^2+^ overload (115). In fact, the Aβ-mediated decrease in zonula occludin-1 (ZO-1) expression is attenuated by neutralizing antibodies against receptor for advanced glycation end-products (RAGE) and inhibitors of calcineurin, suggesting that the Aβ–RAGE interactions disrupt tight junction proteins *via* the Ca^2+^-calcineurin pathway (116).

## Aβ Amyloidosis-Related Retinal Neurodegenerative Diseases

Accumulation and aggregation of Aβ is a common denominator of a number of neurodegenerative diseases. Some of them primarily affect the eye/retina (AMD, glaucoma), while others display more specific cerebral manifestations, such as AD and PD. However, evidence is accumulating in support of retinal alterations that may reflect the cerebral neurodegeneration seen in AD and PD patients.

### Alzheimer’s Disease

Alzheimer’s disease is the main cause of dementia and the most common neurodegenerative disorder in the elderly. It is characterized by cognitive, memory, and language impairments leading to a complete loss of executive functions at the advanced stages (https://www.alz.co.uk/research/WorldAlzheimerReport2015.pdf). From a histophatological point of view, two main hallmarks of AD are Aβ plaques and neurofibrillary tangles (NFTs). The latter are mainly composed of hyperphosphorylated tau protein, a microtubule-associated protein (MAP) essential for the maintenance of neuronal polarity and structure (117). It has been shown that Aβ accumulation leads to disassembly of tau from the microtubules and promotes its hyperphosphorylation (118, 119). The hyperphosphorylation of tau and its subsequent oligomerization results in the formation of intracellular NFTs. Ultimately, cytotoxic NFTs act in synergy with oligomeric Aβ and lead to synaptic dysfunction and axonal loss (120, 121).

#### AD Pathology in the Brain

Functional alterations associated with AD have been extensively studied in the brain at different levels (network/circuit, cellular, subcellular, and molecular) of organization.

##### Synaptic Dysfunction

Amyloid-beta oligomers reduce glutamatergic synaptic transmission by decreasing the number of both AMPA and NMDA postsynaptic receptors (68–71). Besides, a small increase in Aβ has been correlated with increased presynaptic transmission, implicating the activation of α7-nicotinic acetylcholine receptors (nAChR) (32, 122). These synaptic dysfunctions coincide with dysregulation of both LTP and long-term depression (LTD), which are attenuated and enhanced, respectively. Such functional impairments are accompanied with a collapse of dendritic spines and synaptic loss (69, 70, 123). Importantly, AD is characterized by aberrant excitatory network activity and synchronization, which leads to dysfunction of learning and memory circuits and subsequent cognitive decline (124).

##### Mitochondrial Dysfunction and Oxidative Stress

Mitochondrial dysfunction is an early event in AD pathogenesis (87). Both APP (125) and Aβ (126) are targeted to mitochondria. Mitochondrial Aβ accumulation has been clearly demonstrated both in AD patients and in transgenic AD mouse models (127, 128). However, the precise mitochondrial actions of Aβ are still poorly understood. In particular, it is unknown whether mitochondrial translocation of intracellular Aβ is required for the inhibitory effects on mitochondrial membrane potential (MMP) and ATP levels recently demonstrated in a transgenic mouse AD model (TgAPP/PS1) (129). Besides, it has been suggested that Aβ cooperates synergistically with tau in the impairment of oxidative phosphorylation (86). Indeed, several mitochondrial respiratory enzymes were found to be altered in AD, leading to impairments in energy metabolism (130), but the cause–effect relationship between these impairments and Aβ has not been entirely elucidated.

##### Neuroinflammation

Prominent glial cell activation and related neuroinflammation are seen at the advanced stages of AD and likely play a pivotal role in AD progression (100). The aggregation of both Aβ and tau protein leads to the activation of microglia and astroglia, which are consistently found surrounding Aβ deposits in postmortem AD brains (131–133). More recently, positron emission tomography (PET) brought additional *in vivo* evidence for AD-associated cerebral microgliosis (134).

Accordingly, evidence of neuroinflammation was present in all studied AD mouse models (65). In particular, a prominent induction of TNFα and shift from phagocytic M2 toward the cytotoxic-like M1 microglia phenotype has been reported in the hippocampus at the overt stages of AD pathology in TgAPP/PS1 mice, and this effect was reproduced by treating microglia cultures with oligomeric Aβ (135). This upregulation is accompanied by the coincident induction of another major proinflammatory cytokine, IL-1β, not only in the TgAPP/PS1 mouse (136, 137) but also in Tg2576 (138), 3xTg (139), and TgCRND8 (140) mice. Most importantly, all these studies confirmed consistent and concomitant microglia and astrocyte activation.

The microglia M1-like activation state is characterized by uncontrolled proinflammatory cytokine and chemokine secretion, inefficient Aβ phagocytosis, and TLR activation, which further fuels neuroinflammation (65). Among the relevant cytokines and chemokines, monocyte chemoattractant protein (MCP-1) was repeatedly implicated. The membrane pore-forming capacity of Aβ oligomers has also been related to neuroinflammation (141). Classically, deleterious neuroinflammatory environments exacerbate AD-related pathological alterations and have been consistently involved in AD progression. However, evidence is mounting to suggest that neuroinflammation likely also occurs before significant Aβ accumulation (142). Moreover, proinflammatory alterations related to the upregulation of TNFα in the context of partial microglia activation may occur even before Aβ accumulation (143).

##### Amyloid Microangiopathy

Microangiopathy, which comprises a host of pathological alterations in the small blood vessels (arterioles, venules, and capillaries), is closely related to cerebral small vessel disease (CSVD). These are heterogeneous pathological conditions that include cerebral blood flow deregulation, endothelial activation, and blood–brain barrier disruption (144).

Such pathological alterations are also found in cerebral amyloid microangiopathy (145). This particular form of microangiopathy results from Aβ deposition within the walls of capillaries or immediately in the adjacent brain parenchyma (145, 146). According to an emerging concept, these lesions may play a causal role in cerebral dysfunction and precede AD-related cognitive impairments (146). Remarkably, although Aβ accumulates selectively in arterioles, the cortical vasculature network appears to be altered in TgCRND8 mice. Extensive structural and functional alterations were observed, including vessel coiling and looping, increased tortuosity of the venules (but not arterioles), and altered microvascular network cerebral blood flow response to hypercapnia (147).

Another prominent feature of AD-related amyloid microangiopathy is the presence of microbleeds. In the Tg2576 mouse model of AD, these microbleeds are due to leakage or rupture of microvasculature in brain regions affected by vascular amyloid deposits (148). Such microbleeds may be related to the upregulation of BACE1 observed in endothelial cells of the blood–brain barrier in another mouse AD model (43) as well as AD patients (114). The knockdown of miR-195, which regulates BACE1 expression at least in endothelial cells, yields increased tau phosphorylation at Ser202/Thr205, Ser262, Thr231, and Ser422, as well as Cdk5/p25 activation in the rat hippocampus (113).

#### AD Pathology in the Retina

The accumulation of Aβ and its deposition into Aβ plaques have been found in postmortem retinas from AD patients (9). In addition, visual disturbances are common in AD, and they may be due to local retinal abnormalities rather than exclusively related to central, visual cortex alterations (149). However, the molecular mechanisms underlying these visual disturbances and the role that Aβ may play in the retina are still largely unknown. Structural abnormalities identified in retinas of AD patients include reduced number of optic nerve fibers and altered thickness of the parapapillary and macular RNFL (150, 151). These structural changes likely reflect retinal neurodegeneration, such as RGC death (152), and are further associated with optic nerve damage (153).

Consistently, Aβ plaques have also been found in the retina of AD transgenic mouse models (9). Retinas from APP transgenic mouse strains contain 18–70 kDa proteolytic products from APP. The proportion of α-secretase generated C-terminal fragments in transgenic retinas was higher than the fragments generated from β-secretase. However, in ELISA assays, retinal Aβ1–42 was 75 times lower than in transgenic brains and remains undetectable by western blot, indicating that much less Aβ is generated in the retina compared with the brain (154). The age-dependent increase in plaques in the outer and inner plexiform layers (OPL/IPL), INL/ONL, and ganglion cell layer (GCL) (155) coincides only partly with the upregulation of APP, which is seen only in the RGC and INL regions (149). In line with these data, transgenic AD mice display both neuroinflammation and neurodegeneration mostly in the GCL (152, 156), where they correlate with APP induction and Aβ accumulation (149).

Interestingly, a recent study showed that amyloidopathy occurs in the retina prior to the brain in TgAPP/PS1 mice, suggesting that in AD patients, Aβ deposits may also be detected in the retina prior to the brain (10). The study of retinal amyloidopathy may be useful, not only to understand the molecular mechanisms involved in AD but also to search for early-stage AD-related biomarkers. This prospect is even more interesting, considering the possibility of developing a non-invasive method to diagnose early-stage AD through direct retinal imaging.

##### Synaptic Dysfunction in the Retina

Available data concerning AD-related retinal synaptic dysfunctions come exclusively from electroretinogram (ERG) recordings, which give insight into the global electrical response of the retina to a light stimulus. ERGs performed in AD patients at the advanced stages of pathology revealed a significant reduction in the amplitudes of a- and b-waves as well as an increased latency of the response (156, 157). Analogous data have been reported in the aged TgAPP/PS1 mouse model (155). However, while ERG recordings provide a rough estimate of the AD-dependent impairments in glutamate-mediated excitatory neurotransmission in the retina, they do not decipher the underlying mechanisms. Cellular electrophysiology studies (field-recording, patch-clamp) are needed in order to precisely define the neurochemical type of synapses and neurons that are the main targets of Aβ.

##### Neuroinflammation in the Retina

The accumulation of Aβ deposits with age in the retina of a transgenic mouse model of AD is accompanied by an increase in immunoreactivity for MCP-1 and F4/80, which suggests that resident microglia are activated, as well as an increase in terminal deoxynucleotidyl transferase dUTP nick end labeling (TUNEL)-positive profiles in the GCL (149). These results suggest that Aβ-induced neurodegeneration is associated with neuroinflammation (149).

The subretinal microinjection of Aβ yields an adaptive, local inflammatory response, which consists of altered expression patterns of cyclooxygenase-2 (COX-2), glutamine synthetase (GS), inwardly rectifying potassium (Kir) channel Kir4.1, and aquaporin (AQP)-4 water channels in retinal Müller glia cells and of AQP-1 in photoreceptors. Activation of the CCL2/CCR2 chemokine axis, along with microglia activation and migration, is also detectable in this paradigm, whereas its inhibition provides neuroprotection against the deleterious actions of Aβ (158). Moreover, Aβ triggers gliosis characterized by glial fibrillary acidic protein (GFAP), vimentin, and nestin upregulation in Müller cells (159). These alterations are similar to those seen during neuroinflammation in the brain.

The upregulation of GFAP was further confirmed after Aβ injection into the vitreous fluid (160) in both acute (48 h) and delayed (5 months) settings. Remarkably, this study demonstrated a concomitant and selective loss of parvalbumin-expressing neurons in the INL and, to a lower extent, in the GCL (160). The latter finding suggests that, as in the AD brain (161) and transgenic AD mouse models (162, 163), parvalbumin-expressing inhibitory neurons in the retina may be the most vulnerable to Aβ.

##### Mitochondrial Dysfunction and Oxidative Stress in the Retina

The neuroinflammation triggered by subretinal injection of Aβ was accompanied by oxidative stress in the inner and outer retinal segments with an increase in highly reactive unsaturated aldehydes 4-hydroxy 2-non-enal (HNE) and acrolein as well as in 8-hydroxy-2′-deoxyguanosine (8-OHdG), a measure of oxidative damage to DNA (159), which culminated in photoreceptor cell death (158, 159). Accordingly, an inverse approach consisting of intravitreous injection of mitochondrial respiratory complex inhibitors confirmed that inhibition of mitochondrial function and associated oxidative stress resulted in increased APP processing and Aβ accumulation. The latter alterations were also found to be accompanied with GFAP upregulation and glial activation (44).

##### Amyloid Microangiopathy

Amyloid-beta accumulation has been found in the retinal and choroidal vasculature of AD mouse models, suggesting that Aβ may be implicated in alterations in local blood flow (149). Moreover, retinal veins in AD patients are narrowed, and the retinal blood flow is decreased (164). Most importantly, a very large case-controlled study (213 AD patients and 294 cognitively normal controls) of retinal microvasculature networks reported a significant decrease in the branching pattern index (fractal dimension) of the retinal venular tree and arteriolar tortuosity in patients (165). Taken together, recent studies in the brain and retina point to similar alterations in the microvasculature in mouse models and AD patients. Furthermore, retinal microvasculature alterations, accessible to non-invasive imaging, may reflect those occurring in the brain. In line with this assumption, abnormal retinal blood flow has been correlated with degree of cognitive impairment (AD versus MCI versus control subjects), suggesting that blood flow abnormalities may precede AD-related neurodegeneration (166).

### Age-Related Macular Degeneration

Age-related macular degeneration is an age-related retinal degenerative disease that causes irreversible vision loss. It is estimated that up to 50 million people worldwide are affected by AMD, and in western countries 5–10% of individuals over 60 years of age suffer from this disorder (167). AMD is characterized by the build-up of drusen deposits between the Bruch’s membrane (BM) and the RPE, which lead to RPE cell abnormalities, dysfunction of the choroidal blood–eye barrier, and photoreceptor death (168, 169). The most common form of AMD is dry AMD, characterized by thickening of the BM, formation of drusen deposits, and activation of the innate immune response (170). The dry form may progress into the exudative (or wet) form, which is characterized by choroidal neovascularization and retinal edema (171). In some cases, drusen deposits continue to expand and can coalesce, giving rise to the degeneration of a large area of RPE and photoreceptors in a process known as geographic atrophy. Drusen is extracellular deposits composed of different proteins, including Aβ and complement members (172). The mechanism leading to drusen formation is still unclear but may involve the accumulation of toxic by-products of the phototransduction cycle (173). These toxic by-products cause oxidative stress and inflammation, which play a central role in AMD progression (42, 174–177). Drusen-associated amyloidogenic proteins have recently been identified as oligomers (172).

Retinal cells that overlie both soft and hard drusen display numerous structural and molecular abnormalities. Normally detectable only in the outer segments of rod photoreceptors, rod opsin immunolabeling was also observed in the inner segment, cell body, axon, and axon terminal of photoreceptors that overlie drusen (178).

Similar to AD, the risk of developing AMD is also linked to some apolipoprotein E (APOE) polymorphisms. However, in contrast to AD, it has been shown that the e4 allele of the gene encoding APOE is associated with a lower risk of developing AMD, while the e2 allele is associated with a higher risk. Other polymorphisms associated with the development of AMD are linked to genes encoding components of the complement system (170). The polymorphism Y402H in complement factor H (CFH), for example, is the first genetic risk factor for both forms of AMD (179–181). It occurs in 33% of individuals and is associated with a 48% risk for developing AMD (182). CFH is the main inhibitor of the alternative pathway, a key component of the innate immune response. *cfh* KO mice also show features of AMD (183). The mechanisms by which CFH and polymorphisms in the gene affect AMD remain unknown. In 2016, the CFH Y402H polymorphism was identified as a risk factor for AD in a very large cohort of patients (184), confirming previous studies (185).

#### Synaptic Dysfunctions

There is currently no data on putative synaptic dysfunctions in AMD. This may be related to the fact that the main target of neurodegeneration in AMD is the RPE, which is not part of the neuronal network *sensu stricto*. However, RPE cells are excitable, and it would be interesting to explore Aβ-related effects on their excitability.

Drusen-associated abnormalities in the synaptic terminals of photoreceptor neurons have been reported. In AMD-afflicted retinas, but not in normal aged human retinas, a large number of photoreceptor synapses across the entire retina retract into the ONL. This event evokes the subsequent outgrowth of dendrites from postsynaptic bipolar cells, again across the entire retina, and the subsequent rearrangement of synaptic contacts between the photoreceptor and bipolar cells. In addition, an increase in intermediate filament protein immunoreactivity (vimentin and GFAP) is observed within Müller glial cells in areas of the retina overlying drusen. However, other types of retinal neurons (i.e., bipolar, horizontal, amacrine, and ganglion cells) are all, at least structurally, unaffected (186).

#### Mitochondrial Dysfunction and Oxidative Stress

In AMD, the accumulation of lipofuscin, i.e., cross-linked pigmentary deposits from photoreceptor membranes, favors RPE degeneration. Lipofuscin has damaging oxidant properties and has been associated with mitochondrial dysfunction. Similar to what happens within the brain, Aβ accumulation may further exacerbate this state of metabolic and oxidative stress (170). Analogously, Aβ accumulation may contribute to mitochondrial dysfunction in RGCs. Indeed, intracellular Aβ has also been observed in these cells, and it is likely that Aβ interferes with mitochondrial function, following the mechanisms characterized in AD (37).

#### Neuroinflammation

Drusen formation leads to activation of the innate immune system and also to oxidative and metabolic stress, which progressively leads to neurodegeneration. Increased deposition of Aβ has been found in photoreceptor outer segments and in the membrane between the RPE and the BM, in the retinas of both aging humans and mice (51). It has been proposed that along with aging, gradual accumulation of debris may initiate the formation of drusen, which encapsulates different types of proteins, lipids, and inflammatory molecules (176). Among these proteins, extracellular Aβ derived from injured RPE may be included in drusen. Still, the role Aβ plays in this context is unclear. It has been shown that the oligomeric form of Aβ1–42 is implicated in the increased production of ROS, the alteration of RPE cell structure, and transepithelial permeability (91). In addition, Aβ may enhance the release of vascular endothelial growth factor (VEGF) and pigment epithelium-derived factor from RPE cells, favoring angiogenesis (187).

#### Amyloid Microangiopathy

Amyloid microangiopathy has not been extensively studied in AMD. However, it has been proposed that microvascular leakiness may be caused by the promoting effect that amyloidogenesis may exert on neoangiogenesis. VEGF-mediated angiopathy plays a key role in choroidal neovascularization, which is a hallmark of exudative AMD (188). On the other hand, increased VEGF levels may be triggered by members of the complement system, such as C3a and C5a (189). It remains to be determined what triggers the activation of the complement system. Similar to what happens in AD, Aβ may promote its activation (190).

The activated complement system may in turn lead to increased vascular permeability and hypervascularization. This scenario has been experimentally verified in aged Tg2576 mice and postmortem AD brain tissue (191). Neovascularization is a major hallmark of exudative AMD, and by consequence, this form of AMD and AD may share pathological mechanisms in the context of blood–brain barrier impairments. However, a recent study (including 107 individuals diagnosed with AMD) reported no difference between venular and arteriolar calibers in the macula region, at least during the early stages of AMD (192) in agreement with a previous study (193).

### Glaucoma

Glaucoma is a progressive optic neuropathy that represents one of the leading causes of blindness worldwide. It is characterized by the loss of RGC neurons and their axons, with consequent structural changes in the optic nerve and visual field defects. The entire visual pathway, including intracranial optic nerve, lateral geniculate nucleus, and visual cortex, is affected (5, 194, 195). Therefore, glaucoma can be associated with other neurodegenerative disorders, such as AD, since the most vulnerable neuronal target (i.e., RGCs) is common for both pathologies.

One of the major risk factors for developing glaucoma is chronically elevated intraocular pressure (IOP). Accordingly, it has been shown that elevated IOP leads to ganglion cell changes that promote caspase activation and abnormal APP processing (196). Reducing IOP is the only therapy available to limit disease progression; however, the correlation between glaucoma and IOP has only been partially elucidated, and other factors clearly contribute to its pathogenesis (197, 198). Indeed, reducing IOP does not always stop disease progression (199), and some primary open-angle glaucoma patients show normal IOP (200).

It is presently unknown if Aβ is among the additional factors involved in the observed changes in IOP during glaucoma. Nevertheless, Aβ does appear to be a common denominator for glaucoma and AD. Indeed, in glaucoma patients, the level of Aβ in the vitreous fluid is decreased, while tau protein is increased (201). Similarly, in AD patients, the level of Aβ in the cerebrospinal fluid (CSF) is decreased, because of its reduced clearance, whereas tau protein is increased (202). In addition, increased levels of Aβ have been observed in RGCs in rat models of acute ocular hypertension (196, 203). Moreover, inhibiting Aβ production or improving its clearance reduced RGC death (203).

#### Synaptic Dysfunction

Mechanisms of synaptic dysfunction in glaucoma have not yet been investigated.

#### Mitochondrial Dysfunction and Oxidative Stress

Glaucoma has been shown to involve mitochondrial dysfunction (204), and oxidatively modified DNA, proteins, and lipids have been identified in affected patients (205). Importantly, the plasma level of F2-isoprostane lipid was correlated with heat shock protein 72 (HSP72) and heme-oxygenase-1, which are both known to be involved in the defense response against oxidative stress and are increased in glaucoma patients (206).

#### Neuroinflammation

Transcripts of TNFα, IL-2, and IL-6 have been identified in the iris of neovascular glaucoma patients (207). The role of retinal glia-derived proinflammatory cytokines, notably IL-1β and TNFα, in glaucoma has been broadly recognized (208). Important insights into neuroinflammation-related mechanisms of glaucoma have been recently obtained in an elegant study using a rat model of glaucoma. The dominant-negative TNFα inhibitor, XPro1595, which selectively inhibits soluble TNFα, rescued Müller cell and microglia/macrophage activation after induction of ocular hypertension. Moreover, XPro1595 also prevented the TNFα-mediated induction of the Ca^2+^-permeable GluR2 subunit of AMPA glutamate receptors, which are known to be causal in the cytotoxic effects of TNFα, as well as in the death of RGC neurons (209). These data formally demonstrate the causal link between neuroinflammation and neurodegeneration in glaucoma.

#### Amyloid Microangiopathy

To date, putative Aβ-related structural and functional alterations of microvessels have not been investigated in glaucoma. Indeed, a host of publications (more than 2000 referenced in PubMed) deal with hemodynamic alterations that are consistently found in glaucoma (210). However, endothelin-1 and nitric oxide, known to be released by endothelial cells upon activation, are increased in open-angle glaucoma, suggesting the possible involvement of microvasculature in this pathology (210).

## Conclusion

Based on the evidence discussed in this review, it is increasingly clear that, at least in the case of Aβ-amyloidosis, the deleterious effects that Aβ exerts on both cerebral and retinal neurons are very similar. These similarities concern alterations at both the cellular and molecular levels, such as cytokine induction and mitochondrial failure, regardless of the particular disease. Furthermore, Aβ-related alterations, such as oxidative stress, microvasculature abnormalities, and neuroinflammation, are more related to amyloidosis than to the pathological context specific to each disorder (e.g., the different composition of Aβ plaques and drusen in AD and AMD).

Amyloid-beta may therefore be an attractive common target for immunotherapy in both AMD and AD. Encouraging results were obtained after administration of anti-Aβ antibodies in mouse models of AMD (211) and AD (212) that motivated human clinical trials, in spite of some secondary effects. Although the first-generation of Aβ vaccines in AD was interrupted because of severe cerebral hemorrhage (213), new molecules are currently in clinical trials. In particular, GSK933776 was effective in both AMD phase II (214) and AD phase I (215) trials. These clinical data further point to common mechanisms in AD and AMD. Consistently, treatment with an anti-Aβ antibody in a mouse model of AMD yielded a decrease in Aβ deposits both in the retina and the brain (211).

At this stage, many challenges remain for the future. For example, it is of utmost importance to determine whether a coincident oligopathy, such as the PD-associated α-synuclein amyloidosis, may affect Aβ-amyloidosis output in the retina. Understanding whether these two amyloidoses yield additive or synergistic pathological alterations may be very helpful for designing new and more global therapeutic approaches for all relevant diseases.

It is now largely recognized that neurodegenerative alterations in the retina reflect those occurring in the brain, thus raising the hope of using the retina as a source of diagnostic biomarkers for cerebral neurodegeneration. The retina has attracted much interest since, when compared with the brain, it displays the advantage of being relatively less complex structurally and more accessible to non-invasive exploration. Indeed, it may 1 day be possible to use the retina as a proxy to diagnose early neurodegenerative alterations in the brain to target them before neurodegeneration becomes irreversible.

## Author Contributions

AM wrote the first draft of the manuscript, managed the references, and prepared the Figure 2. VD prepared the Figure 1 and brought constructive changes to the text of the manuscript. CC significantly reviewed the text and worked on references indexing. FM made the major modifications in the course of successive reviewing. SK conceived and supervised the preparation of the review.

## Conflict of Interest Statement

The authors declare that the research was conducted in the absence of any commercial or financial relationships that could be construed as a potential conflict of interest. The reviewer AM and handling editor declared their shared affiliation, and the handling editor states that the process nevertheless met the standards of a fair and objective review.

## References

[1] Krantic S Torriglia A. Retina: source of the earliest biomarkers for Alzheimer’s disease? J Alzheimers Dis (2014) 40(2):237–43. 10.3233/JAD-132105 24413614

[2] Nalivaeva NN Belyaev ND Kerridge C Turner AJ. Amyloid-clearing proteins and their epigenetic regulation as a therapeutic target in Alzheimer’s disease. Front Aging Neurosci (2014) 6:235. 10.3389/fnagi.2014.00235 25278875 PMC4166351

[3] Surguchev A Surguchov A. Conformational diseases: looking into the eyes. Brain Res Bull (2010) 81(1):12–24. 10.1016/j.brainresbull.2009.09.015 19808079

[4] Blanks JC Torigoe Y Hinton DR Blanks RH. Retinal pathology in Alzheimer’s disease. I. Ganglion cell loss in foveal/parafoveal retina. Neurobiol Aging (1996) 17(3):377–84. 10.1016/0197-4580(96)00010-3 8725899

[5] Nucci C Martucci A Cesareo M Mancino R Russo R Bagetta G Brain involvement in glaucoma: advanced neuroimaging for understanding and monitoring a new target for therapy. Curr Opin Pharmacol (2013) 13(1):128–33. 10.1016/j.coph.2012.08.004 22981808

[6] Mailankody P Battu R Khanna A Lenka A Yadav R Pal PK. Optical coherence tomography as a tool to evaluate retinal changes in Parkinson’s disease. Parkinsonism Relat Disord (2015) 21(10):1164–9. 10.1016/j.parkreldis.2015.08.002 26297381

[7] Forloni G Artuso V La Vitola P Balducci C. Oligomeropathies and pathogenesis of Alzheimer and Parkinson’s diseases. Mov Disord (2016) 31(6):771–81. 10.1002/mds.26624 27030592

[8] Ho CY Troncoso JC Knox D Stark W Eberhart CG. Beta-amyloid, phospho-tau and alpha-synuclein deposits similar to those in the brain are not identified in the eyes of Alzheimer’s and Parkinson’s disease patients. Brain Pathol (2014) 24(1):25–32. 10.1111/bpa.12070 23714377 PMC3976129

[9] Koronyo-Hamaoui M Koronyo Y Ljubimov AV Miller CA Ko MK Black KL Identification of amyloid plaques in retinas from Alzheimer’s patients and noninvasive in vivo optical imaging of retinal plaques in a mouse model. Neuroimage (2011) 54(Suppl 1):S204–17. 10.1016/j.neuroimage.2010.06.020 20550967 PMC2991559

[10] More SS Vince R. Hyperspectral imaging signatures detect amyloidopathy in Alzheimer’s mouse retina well before onset of cognitive decline. ACS Chem Neurosci (2015) 6(2):306–15. 10.1021/cn500242z 25354367

[11] Bertrand E Lewandowska E Stepien T Szpak GM Pasennik E Modzelewska J. Amyloid angiopathy in idiopathic Parkinson’s disease. Immunohistochemical and ultrastructural study. Folia Neuropathol (2008) 46(4):255–70. 19169967

[12] Chorostecki J Seraji-Bozorgzad N Shah A Bao F Bao G George E Characterization of retinal architecture in Parkinson’s disease. J Neurol Sci (2015) 355(1–2):44–8. 10.1016/j.jns.2015.05.007 26071887

[13] Satue M Seral M Otin S Alarcia R Herrero R Bambo MP Retinal thinning and correlation with functional disability in patients with Parkinson’s disease. Br J Ophthalmol (2014) 98(3):350–5. 10.1136/bjophthalmol-2013-304152 24276697

[14] Huang YM Yin ZQ. Minor retinal degeneration in Parkinson’s disease. Med Hypotheses (2011) 76(2):194–6. 10.1016/j.mehy.2010.09.016 20933338

[15] Jacobsen KT Iverfeldt K. Amyloid precursor protein and its homologues: a family of proteolysis-dependent receptors. Cell Mol Life Sci (2009) 66(14):2299–318. 10.1007/s00018-009-0020-8 19333550 PMC11115575

[16] Niederwolfsgruber E Schmitt TL Blasko I Trieb K Steger MM Maczek C The production of the Alzheimer amyloid precursor protein (APP) in extraneuronal tissue does not increase in old age. J Gerontol A Biol Sci Med Sci (1998) 53(3):B186–90. 10.1093/gerona/53A.3.B186 9597042

[17] Liu X Yu X Zack DJ Zhu H Qian J. TiGER: a database for tissue-specific gene expression and regulation. BMC Bioinformatics (2008) 9:271. 10.1186/1471-2105-9-271 18541026 PMC2438328

[18] Clarris HJ Key B Beyreuther K Masters CL Small DH. Expression of the amyloid protein precursor of Alzheimer’s disease in the developing rat olfactory system. Brain Res Dev Brain Res (1995) 88(1):87–95. 10.1016/0165-3806(95)00083-P 7493410

[19] Apelt J Schliebs R Beck M Rossner S Bigl V. Expression of amyloid precursor protein mRNA isoforms in rat brain is differentially regulated during postnatal maturation and by cholinergic activity. Int J Dev Neurosci (1997) 15(1):95–112. 10.1016/S0736-5748(96)00073-1 9099621

[20] Rohan de Silva HA Jen A Wickenden C Jen LS Wilkinson SL Patel AJ. Cell-specific expression of beta-amyloid precursor protein isoform mRNAs and proteins in neurons and astrocytes. Brain Res Mol Brain Res (1997) 47(1–2):147–56. 10.1016/S0169-328X(97)00045-4 9221912

[21] Yamazaki T Koo EH Selkoe DJ. Trafficking of cell-surface amyloid beta-protein precursor. II. Endocytosis, recycling and lysosomal targeting detected by immunolocalization. J Cell Sci (1996) 109(Pt 5):999–1008. 8743947 10.1242/jcs.109.5.999

[22] Thinakaran G Koo EH. Amyloid precursor protein trafficking, processing, and function. J Biol Chem (2008) 283(44):29615–9. 10.1074/jbc.R800019200 18650430 PMC2573065

[23] Chow VW Mattson MP Wong PC Gleichmann M. An overview of APP processing enzymes and products. Neuromolecular Med (2010) 12(1):1–12. 10.1007/s12017-009-8104-z 20232515 PMC2889200

[24] Fahrenholz F Gilbert S Kojro E Lammich S Postina R. Alpha-secretase activity of the disintegrin metalloprotease ADAM 10. Influences of domain structure. Ann N Y Acad Sci (2000) 920:215–22. 10.1111/j.1749-6632.2000.tb06925.x 11193153

[25] Asai M Hattori C Szabo B Sasagawa N Maruyama K Tanuma S Putative function of ADAM9, ADAM10, and ADAM17 as APP alpha-secretase. Biochem Biophys Res Commun (2003) 301(1):231–5. 10.1016/S0006-291X(02)02999-6 12535668

[26] Tanabe C Hotoda N Sasagawa N Sehara-Fujisawa A Maruyama K Ishiura S. ADAM19 is tightly associated with constitutive Alzheimer’s disease APP alpha-secretase in A172 cells. Biochem Biophys Res Commun (2007) 352(1):111–7. 10.1016/j.bbrc.2006.10.181 17112471

[27] De Strooper B Annaert W. Proteolytic processing and cell biological functions of the amyloid precursor protein. J Cell Sci (2000) 113(Pt 11):1857–70. 10806097 10.1242/jcs.113.11.1857

[28] Wolfe MS Inhibition and modulation of gamma-secretase for Alzheimer’s disease. Neurotherapeutics (2008) 5(3):391–8. 10.1016/j.nurt.2008.05.010 18625450 PMC2572079

[29] Dawkins E Small DH Insights into the physiological function of the beta-amyloid precursor protein: beyond Alzheimer’s disease. J Neurochem (2014) 129(5):756–69. 10.1111/jnc.12675 24517464 PMC4314671

[30] Ring S Weyer SW Kilian SB Waldron E Pietrzik CU Filippov MA The secreted beta-amyloid precursor protein ectodomain APPs alpha is sufficient to rescue the anatomical, behavioral, and electrophysiological abnormalities of APP-deficient mice. J Neurosci (2007) 27(29):7817–26. 10.1523/JNEUROSCI.1026-07.2007 17634375 PMC6672885

[31] Kamenetz F Tomita T Hsieh H Seabrook G Borchelt D Iwatsubo T APP processing and synaptic function. Neuron (2003) 37(6):925–37. 10.1016/S0896-6273(03)00124-7 12670422

[32] Abramov E Dolev I Fogel H Ciccotosto GD Ruff E Slutsky I. Amyloid-beta as a positive endogenous regulator of release probability at hippocampal synapses. Nat Neurosci (2009) 12(12):1567–76. 10.1038/nn.2433 19935655

[33] Yao ZX Papadopoulos V. Function of beta-amyloid in cholesterol transport: a lead to neurotoxicity. FASEB J (2002) 16(12):1677–9. 10.1096/fj.02-0285fje 12206998

[34] Grimm MO Grimm HS Hartmann T. Amyloid beta as a regulator of lipid homeostasis. Trends Mol Med (2007) 13(8):337–44. 10.1016/j.molmed.2007.06.004 17644432

[35] Grimm MO Grimm HS Patzold AJ Zinser EG Halonen R Duering M Regulation of cholesterol and sphingomyelin metabolism by amyloid-beta and presenilin. Nat Cell Biol (2005) 7(11):1118–23. 10.1038/ncb1313 16227967

[36] Lillien L. Neurogenesis in the vertebrate retina. Perspect Dev Neurobiol (1994) 2(2):175–82. 7728501

[37] Chiu K Chan TF Wu A Leung IY So KF Chang RC. Neurodegeneration of the retina in mouse models of Alzheimer’s disease: what can we learn from the retina? Age (Dordr) (2012) 34(3):633–49. 10.1007/s11357-011-9260-2 21559868 PMC3337933

[38] Ratnayaka JA Serpell LC Lotery AJ. Dementia of the eye: the role of amyloid beta in retinal degeneration. Eye (Lond) (2015) 29(8):1013–26. 10.1038/eye.2015.100 26088679 PMC4541342

[39] Dinet V An N Ciccotosto GD Bruban J Maoui A Bellingham SA APP involvement in retinogenesis of mice. Acta Neuropathol (2011) 121(3):351–63. 10.1007/s00401-010-0762-2 20978902

[40] Ho T Vessey KA Cappai R Dinet V Mascarelli F Ciccotosto GD Amyloid precursor protein is required for normal function of the rod and cone pathways in the mouse retina. PLoS One (2012) 7(1):e29892. 10.1371/journal.pone.0029892 22279552 PMC3261162

[41] Morin PJ Abraham CR Amaratunga A Johnson RJ Huber G Sandell JH Amyloid precursor protein is synthesized by retinal ganglion cells, rapidly transported to the optic nerve plasma membrane and nerve terminals, and metabolized. J Neurochem (1993) 61(2):464–73. 10.1111/j.1471-4159.1993.tb02147.x 7687653

[42] Loffler KU Edward DP Tso MO. Immunoreactivity against tau, amyloid precursor protein, and beta-amyloid in the human retina. Invest Ophthalmol Vis Sci (1995) 36(1):24–31. 7822152

[43] Devraj K Poznanovic S Spahn C Schwall G Harter PN Mittelbronn M BACE-1 is expressed in the blood-brain barrier endothelium and is upregulated in a murine model of Alzheimer’s disease. J Cereb Blood Flow Metab (2016) 36(7):1281–94. 10.1177/0271678X15606463 26661166 PMC4929696

[44] Xiong K Cai H Luo XG Struble RG Clough RW Yan XX. Mitochondrial respiratory inhibition and oxidative stress elevate beta-secretase (BACE1) proteins and activity in vivo in the rat retina. Exp Brain Res (2007) 181(3):435–46. 10.1007/s00221-007-0943-y 17429617

[45] Frederikse PH Dubin RA Haynes JI II Piatigorsky J. Structure and alternate tissue-preferred transcription initiation of the mouse alpha B-crystallin/small heat shock protein gene. Nucleic Acids Res (1994) 22(25):5686–94. 10.1093/nar/22.25.5686 7838723 PMC310134

[46] Goldstein LE Muffat JA Cherny RA Moir RD Ericsson MH Huang X Cytosolic beta-amyloid deposition and supranuclear cataracts in lenses from people with Alzheimer’s disease. Lancet (2003) 361(9365):1258–65. 10.1016/S0140-6736(03)12981-9 12699953

[47] Yoneda S Hara H Hirata A Fukushima M Inomata Y Tanihara H. Vitreous fluid levels of beta-amyloid((1-42)) and tau in patients with retinal diseases. Jpn J Ophthalmol (2005) 49(2):106–8. 10.1007/s10384-004-0156-x 15838725

[48] Sipe JD Cohen AS. Review: history of the amyloid fibril. J Struct Biol (2000) 130(2–3):88–98. 10.1006/jsbi.2000.4221 10940217

[49] Marcinkiewicz M Seidah NG. Coordinated expression of beta-amyloid precursor protein and the putative beta-secretase BACE and alpha-secretase ADAM10 in mouse and human brain. J Neurochem (2000) 75(5):2133–43. 10.1046/j.1471-4159.2000.0752133.x 11032903

[50] Fukuchi K Kamino K Deeb SS Smith AC Dang T Martin GM. Overexpression of amyloid precursor protein alters its normal processing and is associated with neurotoxicity. Biochem Biophys Res Commun (1992) 182(1):165–73. 10.1016/S0006-291X(05)80126-3 1731777

[51] Hoh Kam J Lenassi E Jeffery G. Viewing ageing eyes: diverse sites of amyloid Beta accumulation in the ageing mouse retina and the up-regulation of macrophages. PLoS One (2010) 5(10):e13127. 10.1371/journal.pone.0013127 20957206 PMC2948519

[52] Glotin AL Debacq-Chainiaux F Brossas JY Faussat AM Treton J Zubielewicz A Prematurely senescent ARPE-19 cells display features of age-related macular degeneration. Free Radic Biol Med (2008) 44(7):1348–61. 10.1016/j.freeradbiomed.2007.12.023 18226607

[53] Curcio CA Millican CL Allen KA Kalina RE. Aging of the human photoreceptor mosaic: evidence for selective vulnerability of rods in central retina. Invest Ophthalmol Vis Sci (1993) 34(12):3278–96. 8225863

[54] El-Agnaf OM Mahil DS Patel BP Austen BM. Oligomerization and toxicity of beta-amyloid-42 implicated in Alzheimer’s disease. Biochem Biophys Res Commun (2000) 273(3):1003–7. 10.1006/bbrc.2000.3051 10891362

[55] Hardy JA Higgins GA Alzheimer’s disease: the amyloid cascade hypothesis. Science (1992) 256(5054):184–5. 10.1126/science.1566067 1566067

[56] Selkoe DJ Hardy J The amyloid hypothesis of Alzheimer’s disease at 25 years. EMBO Mol Med (2016) 8(6):595–608. 10.15252/emmm.201606210 27025652 PMC4888851

[57] Haass C Selkoe DJ. Soluble protein oligomers in neurodegeneration: lessons from the Alzheimer’s amyloid beta-peptide. Nat Rev Mol Cell Biol (2007) 8(2):101–12. 10.1038/nrm2101 17245412

[58] Mc Donald JM Savva GM Brayne C Welzel AT Forster G Shankar GM The presence of sodium dodecyl sulphate-stable Abeta dimers is strongly associated with Alzheimer-type dementia. Brain (2010) 133(Pt 5):1328–41. 10.1093/brain/awq065 20403962 PMC2859152

[59] Ono K Condron MM Teplow DB. Structure-neurotoxicity relationships of amyloid beta-protein oligomers. Proc Natl Acad Sci U S A (2009) 106(35):14745–50. 10.1073/pnas.0905127106 19706468 PMC2736424

[60] Lesne S Koh MT Kotilinek L Kayed R Glabe CG Yang A A specific amyloid-beta protein assembly in the brain impairs memory. Nature (2006) 440(7082):352–7. 10.1038/nature04533 16541076

[61] Brouillette J The effects of soluble Aβ oligomers on neurodegeneration in Alzheimer’s disease. Curr Pharm Des (2014) 20(15):2506–19. 10.2174/13816128113199990498 23859546

[62] Wirths O Multhaup G Czech C Blanchard V Moussaoui S Tremp G Intraneuronal Abeta accumulation precedes plaque formation in beta-amyloid precursor protein and presenilin-1 double-transgenic mice. Neurosci Lett (2001) 306(1–2):116–20. 10.1016/S0304-3940(01)01876-6 11403971

[63] Gomez-Ramos P Asuncion Moran M. Ultrastructural localization of intraneuronal Abeta-peptide in Alzheimer disease brains. J Alzheimers Dis (2007) 11(1):53–9. 17361035 10.3233/jad-2007-11109

[64] Shankar GM Li S Mehta TH Garcia-Munoz A Shepardson NE Smith I Amyloid-beta protein dimers isolated directly from Alzheimer’s brains impair synaptic plasticity and memory. Nat Med (2008) 14(8):837–42. 10.1038/nm1782 18568035 PMC2772133

[65] Minter MR Taylor JM Crack PJ. The contribution of neuroinflammation to amyloid toxicity in Alzheimer’s disease. J Neurochem (2016) 136(3):457–74. 10.1111/jnc.13411 26509334

[66] Van Nostrand WE The influence of the amyloid ss-protein and its precursor in modulating cerebral hemostasis. Biochim Biophys Acta (2016) 1862(5):1018–26. 10.1016/j.bbadis.2015.10.020 26519139 PMC4821744

[67] Palop JJ Mucke L. Amyloid-beta-induced neuronal dysfunction in Alzheimer’s disease: from synapses toward neural networks. Nat Neurosci (2010) 13(7):812–8. 10.1038/nn.2583 20581818 PMC3072750

[68] Hsia AY Masliah E McConlogue L Yu GQ Tatsuno G Hu K Plaque-independent disruption of neural circuits in Alzheimer’s disease mouse models. Proc Natl Acad Sci U S A (1999) 96(6):3228–33. 10.1073/pnas.96.6.3228 10077666 PMC15924

[69] Walsh DM Klyubin I Fadeeva JV Cullen WK Anwyl R Wolfe MS Naturally secreted oligomers of amyloid beta protein potently inhibit hippocampal long-term potentiation in vivo. Nature (2002) 416(6880):535–9. 10.1038/416535a 11932745

[70] Hsieh H Boehm J Sato C Iwatsubo T Tomita T Sisodia S AMPAR removal underlies Abeta-induced synaptic depression and dendritic spine loss. Neuron (2006) 52(5):831–43. 10.1016/j.neuron.2006.10.035 17145504 PMC1850952

[71] Shankar GM Bloodgood BL Townsend M Walsh DM Selkoe DJ Sabatini BL. Natural oligomers of the Alzheimer amyloid-beta protein induce reversible synapse loss by modulating an NMDA-type glutamate receptor-dependent signaling pathway. J Neurosci (2007) 27(11):2866–75. 10.1523/JNEUROSCI.4970-06.2007 17360908 PMC6672572

[72] Liu SJ Gasperini R Foa L Small DH. Amyloid-beta decreases cell-surface AMPA receptors by increasing intracellular calcium and phosphorylation of GluR2. J Alzheimers Dis (2010) 21(2):655–66. 10.3233/JAD-2010-091654 20571220

[73] Snyder EM Nong Y Almeida CG Paul S Moran T Choi EY Regulation of NMDA receptor trafficking by amyloid-beta. Nat Neurosci (2005) 8(8):1051–8. 10.1038/nn1503 16025111

[74] Puzzo D Privitera L Leznik E Fa M Staniszewski A Palmeri A Picomolar amyloid-beta positively modulates synaptic plasticity and memory in hippocampus. J Neurosci (2008) 28(53):14537–45. 10.1523/JNEUROSCI.2692-08.2008 19118188 PMC2673049

[75] Vezzani A Granata T. Brain inflammation in epilepsy: experimental and clinical evidence. Epilepsia (2005) 46(11):1724–43. 10.1111/j.1528-1167.2005.00298.x 16302852

[76] Minkeviciene R Rheims S Dobszay MB Zilberter M Hartikainen J Fulop L Amyloid beta-induced neuronal hyperexcitability triggers progressive epilepsy. J Neurosci (2009) 29(11):3453–62. 10.1523/JNEUROSCI.5215-08.2009 19295151 PMC6665248

[77] Tamagnini F Scullion S Brown JT Randall AD Intrinsic excitability changes induced by acute treatment of hippocampal CA1 pyramidal neurons with exogenous amyloid beta peptide. Hippocampus (2015) 25(7):786–97. 10.1002/hipo.22403 25515596 PMC4791149

[78] Walsh DT Bresciani L Saunders D Manca MF Jen A Gentleman SM Amyloid beta peptide causes chronic glial cell activation and neuro-degeneration after intravitreal injection. Neuropathol Appl Neurobiol (2005) 31(5):491–502. 10.1111/j.1365-2990.2005.00666.x 16150120

[79] Aruoma OI Jen SS Watts HR George J Gentleman SM Anderson PJ Acute and chronic effects of intravitreally injected beta-amyloid on the neurotransmitter system in the retina. Toxicology (2009) 256(1–2):92–100. 10.1016/j.tox.2008.11.007 19059454 PMC6548329

[80] Kumar A Singh A. A review on mitochondrial restorative mechanism of antioxidants in Alzheimer’s disease and other neurological conditions. Front Pharmacol (2015) 6:206. 10.3389/fphar.2015.00206 26441662 PMC4585235

[81] Ohyagi Y Asahara H Chui DH Tsuruta Y Sakae N Miyoshi K Intracellular Abeta42 activates p53 promoter: a pathway to neurodegeneration in Alzheimer’s disease. FASEB J (2005) 19(2):255–7. 10.1096/fj.04-2637fje 15548589

[82] Suo Z Cox AA Bartelli N Rasul I Festoff BW Premont RT GRK5 deficiency leads to early Alzheimer-like pathology and working memory impairment. Neurobiol Aging (2007) 28(12):1873–88. 10.1016/j.neurobiolaging.2006.08.013 17011668

[83] Sonkusare SK Kaul CL Ramarao P Dementia of Alzheimer’s disease and other neurodegenerative disorders – memantine, a new hope. Pharmacol Res (2005) 51(1):1–17. 10.1016/j.phrs.2004.05.005 15519530

[84] Huang HM Zhang H Xu H Gibson GE. Inhibition of the alpha-ketoglutarate dehydrogenase complex alters mitochondrial function and cellular calcium regulation. Biochim Biophys Acta (2003) 1637(1):119–26. 10.1016/S0925-4439(02)00222-3 12527416

[85] Bubber P Haroutunian V Fisch G Blass JP Gibson GE. Mitochondrial abnormalities in Alzheimer brain: mechanistic implications. Ann Neurol (2005) 57(5):695–703. 10.1002/ana.20474 15852400

[86] Rhein V Song X Wiesner A Ittner LM Baysang G Meier F Amyloid-beta and tau synergistically impair the oxidative phosphorylation system in triple transgenic Alzheimer’s disease mice. Proc Natl Acad Sci U S A (2009) 106(47):20057–62. 10.1073/pnas.0905529106 19897719 PMC2774257

[87] Swerdlow RH Burns JM Khan SM. The Alzheimer’s disease mitochondrial cascade hypothesis. J Alzheimers Dis (2010) 20(Suppl 2):S265–79. 10.3233/JAD-2010-100339 20442494 PMC2883665

[88] Lustbader JW Cirilli M Lin C Xu HW Takuma K Wang N ABAD directly links Abeta to mitochondrial toxicity in Alzheimer’s disease. Science (2004) 304(5669):448–52. 10.1126/science.1091230 15087549

[89] Calkins MJ Reddy PH. Amyloid beta impairs mitochondrial anterograde transport and degenerates synapses in Alzheimer’s disease neurons. Biochim Biophys Acta (2011) 1812(4):507–13. 10.1016/j.bbadis.2011.01.007 21241801 PMC3042500

[90] Manczak M Calkins MJ Reddy PH. Impaired mitochondrial dynamics and abnormal interaction of amyloid beta with mitochondrial protein Drp1 in neurons from patients with Alzheimer’s disease: implications for neuronal damage. Hum Mol Genet (2011) 20(13):2495–509. 10.1093/hmg/ddr139 21459773 PMC3109997

[91] Bruban J Glotin AL Dinet V Chalour N Sennlaub F Jonet L Amyloid-beta(1-42) alters structure and function of retinal pigmented epithelial cells. Aging Cell (2009) 8(2):162–77. 10.1111/j.1474-9726.2009.00456.x 19239420

[92] De Strooper B Karran E. The cellular phase of Alzheimer’s disease. Cell (2016) 164(4):603–15. 10.1016/j.cell.2015.12.056 26871627

[93] Lian H Yang L Cole A Sun L Chiang AC Fowler SW NFkappaB-activated astroglial release of complement C3 compromises neuronal morphology and function associated with Alzheimer’s disease. Neuron (2015) 85(1):101–15. 10.1016/j.neuron.2014.11.018 25533482 PMC4289109

[94] Lian H Litvinchuk A Chiang AC Aithmitti N Jankowsky JL Zheng H. Astrocyte-microglia cross talk through complement activation modulates amyloid pathology in mouse models of Alzheimer’s disease. J Neurosci (2016) 36(2):577–89. 10.1523/JNEUROSCI.2117-15.2016 26758846 PMC4710776

[95] Osborn LM Kamphuis W Wadman WJ Hol EM. Astrogliosis: an integral player in the pathogenesis of Alzheimer’s disease. Prog Neurobiol (2016) S0301-0082(15):30021–6. 10.1016/j.pneurobio.2016.01.001 26797041

[96] Pihlaja R Koistinaho J Kauppinen R Sandholm J Tanila H Koistinaho M Multiple cellular and molecular mechanisms are involved in human Abeta clearance by transplanted adult astrocytes. Glia (2011) 59(11):1643–57. 10.1002/glia.21212 21826742

[97] Saido T Leissring MA Proteolytic degradation of amyloid beta-protein. Cold Spring Harb Perspect Med (2012) 2(6):a006379 10.1101/cshperspect.a006379 22675659 PMC3367539

[98] Kettenmann H Hanisch UK Noda M Verkhratsky A. Physiology of microglia. Physiol Rev (2011) 91(2):461–553. 10.1152/physrev.00011.2010 21527731

[99] Ji K Akgul G Wollmuth LP Tsirka SE. Microglia actively regulate the number of functional synapses. PLoS One (2013) 8(2):e56293. 10.1371/journal.pone.0056293 23393609 PMC3564799

[100] Heneka MT Carson MJ El Khoury J Landreth GE Brosseron F Feinstein DL Neuroinflammation in Alzheimer’s disease. Lancet Neurol (2015) 14(4):388–405. 10.1016/S1474-4422(15)70016-5 25792098 PMC5909703

[101] El Khoury JB Moore KJ Means TK Leung J Terada K Toft M CD36 mediates the innate host response to beta-amyloid. J Exp Med (2003) 197(12):1657–66. 10.1084/jem.20021546 12796468 PMC2193948

[102] Stewart CR Stuart LM Wilkinson K van Gils JM Deng J Halle A CD36 ligands promote sterile inflammation through assembly of a toll-like receptor 4 and 6 heterodimer. Nat Immunol (2010) 11(2):155–61. 10.1038/ni.1836 20037584 PMC2809046

[103] Liu Y Walter S Stagi M Cherny D Letiembre M Schulz-Schaeffer W LPS receptor (CD14): a receptor for phagocytosis of Alzheimer’s amyloid peptide. Brain (2005) 128(Pt 8):1778–89. 10.1093/brain/awh531 15857927

[104] Lee CY Landreth GE. The role of microglia in amyloid clearance from the AD brain. J Neural Transm (Vienna) (2010) 117(8):949–60. 10.1007/s00702-010-0433-4 20552234 PMC3653296

[105] Lue LF Rydel R Brigham EF Yang LB Hampel H Murphy GM Jr Inflammatory repertoire of Alzheimer’s disease and nondemented elderly microglia in vitro. Glia (2001) 35(1):72–9. 10.1002/glia.1072 11424194

[106] Patel NS Paris D Mathura V Quadros AN Crawford FC Mullan MJ. Inflammatory cytokine levels correlate with amyloid load in transgenic mouse models of Alzheimer’s disease. J Neuroinflammation (2005) 2(1):9. 10.1186/1742-2094-2-9 15762998 PMC555557

[107] Liao YF Wang BJ Cheng HT Kuo LH Wolfe MS. Tumor necrosis factor-alpha, interleukin-1beta, and interferon-gamma stimulate gamma-secretase-mediated cleavage of amyloid precursor protein through a JNK-dependent MAPK pathway. J Biol Chem (2004) 279(47):49523–32. 10.1074/jbc.M402034200 15347683

[108] Yamamoto M Kiyota T Horiba M Buescher JL Walsh SM Gendelman HE Interferon-gamma and tumor necrosis factor-alpha regulate amyloid-beta plaque deposition and beta-secretase expression in Swedish mutant APP transgenic mice. Am J Pathol (2007) 170(2):680–92. 10.2353/ajpath.2007.060378 17255335 PMC1851864

[109] He P Zhong Z Lindholm K Berning L Lee W Lemere C Deletion of tumor necrosis factor death receptor inhibits amyloid beta generation and prevents learning and memory deficits in Alzheimer’s mice. J Cell Biol (2007) 178(5):829–41. 10.1083/jcb.200705042 17724122 PMC2064547

[110] Chen ST Gentleman SM Garey LJ Jen LS. Distribution of beta-amyloid precursor and B-cell lymphoma protooncogene proteins in the rat retina after optic nerve transection or vascular lesion. J Neuropathol Exp Neurol (1996) 55(10):1073–82. 10.1097/00005072-199655100-00007 8858004

[111] Cunvong K Huffmire D Ethell DW Cameron DJ Amyloid-beta increases capillary bed density in the adult zebrafish retina. Invest Ophthalmol Vis Sci (2013) 54(2):1516–21. 10.1167/iovs.12-10821 23404118

[112] Cai J Qi X Kociok N Skosyrski S Emilio A Ruan Q β-Secretase (BACE1) inhibition causes retinal pathology by vascular dysregulation and accumulation of age pigment. EMBO Mol Med (2012) 4(9):980–91. 10.1002/emmm.201101084 22903875 PMC3491829

[113] Sun LH Ban T Liu CD Chen QX Wang X Yan ML Activation of Cdk5/p25 and tau phosphorylation following chronic brain hypoperfusion in rats involves microRNA-195 down-regulation. J Neurochem (2015) 134(6):1139–51. 10.1111/jnc.13212 26118667

[114] Cheng X He P Yao H Dong Q Li R Shen Y. Occludin deficiency with BACE1 elevation in cerebral amyloid angiopathy. Neurology (2014) 82(19):1707–15. 10.1212/WNL.0000000000000403 24739782 PMC4032211

[115] Koizumi K Wang G Park L Endothelial dysfunction and amyloid-beta-induced neurovascular alterations. Cell Mol Neurobiol (2016) 36(2):155–65. 10.1007/s10571-015-0256-9 26328781 PMC4775455

[116] Kook SY Seok Hong H Moon M Mook-Jung I. Disruption of blood-brain barrier in Alzheimer disease pathogenesis. Tissue Barriers (2013) 1(2):e23993. 10.4161/tisb.23993 24665385 PMC3887048

[117] Caceres A Kosik KS. Inhibition of neurite polarity by tau antisense oligonucleotides in primary cerebellar neurons. Nature (1990) 343(6257):461–3. 10.1038/343461a0 2105469

[118] King ME Kan HM Baas PW Erisir A Glabe CG Bloom GS. Tau-dependent microtubule disassembly initiated by prefibrillar beta-amyloid. J Cell Biol (2006) 175(4):541–6. 10.1083/jcb.200605187 17101697 PMC2064590

[119] Khan SS Bloom GS. Tau: the center of a signaling nexus in Alzheimer’s disease. Front Neurosci (2016) 10:31. 10.3389/fnins.2016.00031 26903798 PMC4746348

[120] Brion JP. The role of neurofibrillary tangles in Alzheimer disease. Acta Neurol Belg (1998) 98(2):165–74. 9686275

[121] Sorrentino G Bonavita V. Neurodegeneration and Alzheimer’s disease: the lesson from tauopathies. Neurol Sci (2007) 28(2):63–71. 10.1007/s10072-007-0789-x 17464468

[122] Dineley KT Bell KA Bui D Sweatt JD. beta-Amyloid peptide activates alpha 7 nicotinic acetylcholine receptors expressed in *Xenopus* oocytes. J Biol Chem (2002) 277(28):25056–61. 10.1074/jbc.M200066200 11983690

[123] Li S Hong S Shepardson NE Walsh DM Shankar GM Selkoe D. Soluble oligomers of amyloid beta protein facilitate hippocampal long-term depression by disrupting neuronal glutamate uptake. Neuron (2009) 62(6):788–801. 10.1016/j.neuron.2009.05.012 19555648 PMC2702854

[124] Palop JJ Chin J Roberson ED Wang J Thwin MT Bien-Ly N Aberrant excitatory neuronal activity and compensatory remodeling of inhibitory hippocampal circuits in mouse models of Alzheimer’s disease. Neuron (2007) 55(5):697–711. 10.1016/j.neuron.2007.07.025 17785178 PMC8055171

[125] Anandatheerthavarada HK Biswas G Robin MA Avadhani NG. Mitochondrial targeting and a novel transmembrane arrest of Alzheimer’s amyloid precursor protein impairs mitochondrial function in neuronal cells. J Cell Biol (2003) 161(1):41–54. 10.1083/jcb.200207030 12695498 PMC2172865

[126] Pinho CM Teixeira PF Glaser E Mitochondrial import and degradation of amyloid-beta peptide. Biochim Biophys Acta (2014) 1837(7):1069–74. 10.1016/j.bbabio.2014.02.007 24561226

[127] Chen JX Yan SD. Amyloid-beta-induced mitochondrial dysfunction. J Alzheimers Dis (2007) 12(2):177–84. 17917162 10.3233/jad-2007-12208 PMC3687350

[128] Picone P Nuzzo D Caruana L Scafidi V Di Carlo M. Mitochondrial dysfunction: different routes to Alzheimer’s disease therapy. Oxid Med Cell Longev (2014) 2014:780179. 10.1155/2014/780179 25221640 PMC4158152

[129] Ren H Fu K Wang D Mu C Wang G. Oxidized DJ-1 interacts with the mitochondrial protein BCL-XL. J Biol Chem (2011) 286(40):35308–17. 10.1074/jbc.M110.207134 21852238 PMC3186373

[130] Moreira PI Carvalho C Zhu X Smith MA Perry G. Mitochondrial dysfunction is a trigger of Alzheimer’s disease pathophysiology. Biochim Biophys Acta (2010) 1802(1):2–10. 10.1016/j.bbadis.2009.10.006 19853658

[131] Beach TG Walker R McGeer EG. Patterns of gliosis in Alzheimer’s disease and aging cerebrum. Glia (1989) 2(6):420–36. 10.1002/glia.440020605 2531723

[132] Delacourte A. General and dramatic glial reaction in Alzheimer brains. Neurology (1990) 40(1):33–7. 10.1212/WNL.40.1.33 2296379

[133] Arends YM Duyckaerts C Rozemuller JM Eikelenboom P Hauw JJ. Microglia, amyloid and dementia in Alzheimer disease. A correlative study. Neurobiol Aging (2000) 21(1):39–47. 10.1016/S0197-4580(00)00094-4 10794847

[134] Cagnin A Brooks DJ Kennedy AM Gunn RN Myers R Turkheimer FE In-vivo measurement of activated microglia in dementia. Lancet (2001) 358(9280):461–7. 10.1016/S0140-6736(01)05625-2 11513911

[135] Jimenez S Baglietto-Vargas D Caballero C Moreno-Gonzalez I Torres M Sanchez-Varo R Inflammatory response in the hippocampus of PS1M146L/APP751SL mouse model of Alzheimer’s disease: age-dependent switch in the microglial phenotype from alternative to classic. J Neurosci (2008) 28(45):11650–61. 10.1523/JNEUROSCI.3024-08.2008 18987201 PMC6671312

[136] Couturier J Paccalin M Morel M Terro F Milin S Pontcharraud R Prevention of the beta-amyloid peptide-induced inflammatory process by inhibition of double-stranded RNA-dependent protein kinase in primary murine mixed co-cultures. J Neuroinflammation (2011) 8:72 10.1186/1742-2094-8-72 21699726 PMC3131234

[137] Zhang W Bai M Xi Y Hao J Zhang Z Su C Multiple inflammatory pathways are involved in the development and progression of cognitive deficits in APPswe/PS1dE9 mice. Neurobiol Aging (2012) 33(11):2661–77. 10.1016/j.neurobiolaging.2011.12.023 22277264

[138] Apelt J Schliebs R. Beta-amyloid-induced glial expression of both pro- and anti-inflammatory cytokines in cerebral cortex of aged transgenic Tg2576 mice with Alzheimer plaque pathology. Brain Res (2001) 894(1):21–30. 10.1016/S0006-8993(00)03176-0 11245811

[139] Janelsins MC Mastrangelo MA Oddo S LaFerla FM Federoff HJ Bowers WJ. Early correlation of microglial activation with enhanced tumor necrosis factor-alpha and monocyte chemoattractant protein-1 expression specifically within the entorhinal cortex of triple transgenic Alzheimer’s disease mice. J Neuroinflammation (2005) 2:23. 10.1186/1742-2094-2-23 16232318 PMC1276812

[140] Ma K Mount HT McLaurin J Region-specific distribution of beta-amyloid peptide and cytokine expression in TgCRND8 mice. Neurosci Lett (2011) 492(1):5–10. 10.1016/j.neulet.2011.01.035 21295112

[141] Fernandez-Perez EJ Peters C Aguayo LG. Membrane damage induced by amyloid beta and a potential link with neuroinflammation. Curr Pharm Des (2016) 22(10):1295–304. 10.2174/138161282210160304111702 26972288

[142] Wright AL Zinn R Hohensinn B Konen LM Beynon SB Tan RP Neuroinflammation and neuronal loss precede Abeta plaque deposition in the hAPP-J20 mouse model of Alzheimer’s disease. PLoS One (2013) 8(4):e59586 10.1371/journal.pone.0059586 23560052 PMC3613362

[143] Cavanagh C Colby-Milley J Bouvier D Farso M Chabot JG Quirion R βCTF-correlated burst of hippocampal TNFalpha occurs at a very early, pre-plaque stage in the TgCRND8 mouse model of Alzheimer’s disease. J Alzheimers Dis (2013) 36(2):233–8. 10.3233/JAD-122131 23579326

[144] Hainsworth AH Oommen AT Bridges LR. Endothelial cells and human cerebral small vessel disease. Brain Pathol (2015) 25(1):44–50. 10.1111/bpa.12224 25521176 PMC8029339

[145] Yamada M. Cerebral amyloid angiopathy: emerging concepts. J Stroke (2015) 17(1):17–30. 10.5853/jos.2015.17.1.17 25692104 PMC4325636

[146] Vinters HV. Emerging concepts in Alzheimer’s disease. Annu Rev Pathol (2015) 10:291–319. 10.1146/annurev-pathol-020712-163927 25387055

[147] Lai AY Dorr A Thomason LA Koletar MM Sled JG Stefanovic B Venular degeneration leads to vascular dysfunction in a transgenic model of Alzheimer’s disease. Brain (2015) 138(Pt 4):1046–58. 10.1093/brain/awv023 25688079

[148] Lo P Crouzet C Vasilevko V Choi B. Visualization of microbleeds with optical histology in mouse model of cerebral amyloid angiopathy. Microvasc Res (2016) 105:109–13. 10.1016/j.mvr.2016.02.002 26876114 PMC4814270

[149] Ning A Cui J To E Ashe KH Matsubara J. Amyloid-beta deposits lead to retinal degeneration in a mouse model of Alzheimer disease. Invest Ophthalmol Vis Sci (2008) 49(11):5136–43. 10.1167/iovs.08-1849 18566467 PMC3947384

[150] Danesh-Meyer HV Birch H Ku JY Carroll S Gamble G. Reduction of optic nerve fibers in patients with Alzheimer disease identified by laser imaging. Neurology (2006) 67(10):1852–4. 10.1212/01.wnl.0000244490.07925.8b 17130422

[151] Paquet C Boissonnot M Roger F Dighiero P Gil R Hugon J. Abnormal retinal thickness in patients with mild cognitive impairment and Alzheimer’s disease. Neurosci Lett (2007) 420(2):97–9. 10.1016/j.neulet.2007.02.090 17543991

[152] Blanks JC Hinton DR Sadun AA Miller CA. Retinal ganglion cell degeneration in Alzheimer’s disease. Brain Res (1989) 501(2):364–72. 10.1016/0006-8993(89)90653-7 2819446

[153] Sadun AA Bassi CJ. Optic nerve damage in Alzheimer’s disease. Ophthalmology (1990) 97(1):9–17. 10.1016/S0161-6420(90)32621-0 2314849

[154] Dutescu RM Li QX Crowston J Masters CL Baird PN Culvenor JG. Amyloid precursor protein processing and retinal pathology in mouse models of Alzheimer’s disease. Graefes Arch Clin Exp Ophthalmol (2009) 247(9):1213–21. 10.1007/s00417-009-1060-3 19271231

[155] Perez SE Lumayag S Kovacs B Mufson EJ Xu S. Beta-amyloid deposition and functional impairment in the retina of the APPswe/PS1DeltaE9 transgenic mouse model of Alzheimer’s disease. Invest Ophthalmol Vis Sci (2009) 50(2):793–800. 10.1167/iovs.08-2384 18791173 PMC3697019

[156] Parnell M Guo L Abdi M Cordeiro MF. Ocular manifestations of Alzheimer’s disease in animal models. Int J Alzheimers Dis (2012) 2012:786494. 10.1155/2012/786494 22666623 PMC3362039

[157] Parisi V Restuccia R Fattapposta F Mina C Bucci MG Pierelli F. Morphological and functional retinal impairment in Alzheimer’s disease patients. Clin Neurophysiol (2001) 112(10):1860–7. 10.1016/S1388-2457(01)00620-4 11595144

[158] Bruban J Maoui A Chalour N An N Jonet L Feumi C CCR2/CCL2-mediated inflammation protects photoreceptor cells from amyloid-beta-induced apoptosis. Neurobiol Dis (2011) 42(1):55–72. 10.1016/j.nbd.2011.01.004 21220018

[159] Dinet V Bruban J Chalour N Maoui A An N Jonet L Distinct effects of inflammation on gliosis, osmohomeostasis, and vascular integrity during amyloid beta-induced retinal degeneration. Aging Cell (2012) 11(4):683–93. 10.1111/j.1474-9726.2012.00834.x 22577879

[160] Walsh DT Montero RM Bresciani LG Jen AY Leclercq PD Saunders D Amyloid-beta peptide is toxic to neurons in vivo via indirect mechanisms. Neurobiol Dis (2002) 10(1):20–7. 10.1006/nbdi.2002.0485 12079400

[161] Solodkin A Veldhuizen SD Van Hoesen GW. Contingent vulnerability of entorhinal parvalbumin-containing neurons in Alzheimer’s disease. J Neurosci (1996) 16(10):3311–21. 8627368 10.1523/JNEUROSCI.16-10-03311.1996 PMC6579156

[162] Takahashi H Brasnjevic I Rutten BP Van Der Kolk N Perl DP Bouras C Hippocampal interneuron loss in an APP/PS1 double mutant mouse and in Alzheimer’s disease. Brain Struct Funct (2010) 214(2–3):145–60. 10.1007/s00429-010-0242-4 20213270 PMC3038332

[163] Albuquerque MS Mahar I Davoli MA Chabot JG Mechawar N Quirion R Regional and sub-regional differences in hippocampal GABAergic neuronal vulnerability in the TgCRND8 mouse model of Alzheimer’s disease. Front Aging Neurosci (2015) 7:30. 10.3389/fnagi.2015.00030 25852545 PMC4371759

[164] Berisha F Feke GT Trempe CL McMeel JW Schepens CL. Retinal abnormalities in early Alzheimer’s disease. Invest Ophthalmol Vis Sci (2007) 48(5):2285–9. 10.1167/iovs.06-1029 17460292

[165] Williams MA McGowan AJ Cardwell CR Cheung CY Craig D Passmore P Retinal microvascular network attenuation in Alzheimer’s disease. Alzheimers Dement (Amst) (2015) 1(2):229–35. 10.1016/j.dadm.2015.04.001 26634224 PMC4629099

[166] Feke GT Hyman BT Stern RA Pasquale LR. Retinal blood flow in mild cognitive impairment and Alzheimer’s disease. Alzheimers Dement (Amst) (2015) 1(2):144–51. 10.1016/j.dadm.2015.01.004 27239502 PMC4876882

[167] Klein R Peto T Bird A Vannewkirk MR. The epidemiology of age-related macular degeneration. Am J Ophthalmol (2004) 137(3):486–95. 10.1016/j.ajo.2003.11.069 15013873

[168] McLeod DS Taomoto M Otsuji T Green WR Sunness JS Lutty GA. Quantifying changes in RPE and choroidal vasculature in eyes with age-related macular degeneration. Invest Ophthalmol Vis Sci (2002) 43(6):1986–93. 12037009

[169] Zarbin MA. Current concepts in the pathogenesis of age-related macular degeneration. Arch Ophthalmol (2004) 122(4):598–614. 10.1001/archopht.122.4.598 15078679

[170] Sivak JM. The aging eye: common degenerative mechanisms between the Alzheimer’s brain and retinal disease. Invest Ophthalmol Vis Sci (2013) 54(1):871–80. 10.1167/iovs.12-10827 23364356

[171] de Jong PT Age-related macular degeneration. N Engl J Med (2006) 355(14):1474–85. 10.1056/NEJMra062326 17021323

[172] Luibl V Isas JM Kayed R Glabe CG Langen R Chen J. Drusen deposits associated with aging and age-related macular degeneration contain nonfibrillar amyloid oligomers. J Clin Invest (2006) 116(2):378–85. 10.1172/JCI25843 16453022 PMC1359048

[173] Sparrow JR. Bisretinoids of RPE lipofuscin: trigger for complement activation in age-related macular degeneration. Adv Exp Med Biol (2010) 703:63–74. 10.1007/978-1-4419-5635-4_5 20711707

[174] Johnson LV Leitner WP Rivest AJ Staples MK Radeke MJ Anderson DH. The Alzheimer’s A beta-peptide is deposited at sites of complement activation in pathologic deposits associated with aging and age-related macular degeneration. Proc Natl Acad Sci U S A (2002) 99(18):11830–5. 10.1073/pnas.192203399 12189211 PMC129354

[175] Dentchev T Milam AH Lee VM Trojanowski JQ Dunaief JL. Amyloid-beta is found in drusen from some age-related macular degeneration retinas, but not in drusen from normal retinas. Mol Vis (2003) 9:184–90. 12764254

[176] Anderson DH Talaga KC Rivest AJ Barron E Hageman GS Johnson LV. Characterization of beta amyloid assemblies in drusen: the deposits associated with aging and age-related macular degeneration. Exp Eye Res (2004) 78(2):243–56. 10.1016/j.exer.2003.10.011 14729357

[177] Glabe CG. Common mechanisms of amyloid oligomer pathogenesis in degenerative disease. Neurobiol Aging (2006) 27(4):570–5. 10.1016/j.neurobiolaging.2005.04.017 16481071

[178] Johnson PT Lewis GP Talaga KC Brown MN Kappel PJ Fisher SK Drusen-associated degeneration in the retina. Invest Ophthalmol Vis Sci (2003) 44(10):4481–8. 10.1167/iovs.03-0436 14507896

[179] Edwards AO Ritter R III Abel KJ Manning A Panhuysen C Farrer LA. Complement factor H polymorphism and age-related macular degeneration. Science (2005) 308(5720):421–4. 10.1126/science.1110189 15761121

[180] Hageman GS Anderson DH Johnson LV Hancox LS Taiber AJ Hardisty LI A common haplotype in the complement regulatory gene factor H (HF1/CFH) predisposes individuals to age-related macular degeneration. Proc Natl Acad Sci U S A (2005) 102(20):7227–32. 10.1073/pnas.0501536102 15870199 PMC1088171

[181] Haines JL Hauser MA Schmidt S Scott WK Olson LM Gallins P Complement factor H variant increases the risk of age-related macular degeneration. Science (2005) 308(5720):419–21. 10.1126/science.1110359 15761120

[182] Despriet DD Klaver CC Witteman JC Bergen AA Kardys I de Maat MP Complement factor H polymorphism, complement activators, and risk of age-related macular degeneration. JAMA (2006) 296(3):301–9. 10.1001/jama.296.3.301 16849663

[183] Coffey PJ Gias C McDermott CJ Lundh P Pickering MC Sethi C Complement factor H deficiency in aged mice causes retinal abnormalities and visual dysfunction. Proc Natl Acad Sci U S A (2007) 104(42):16651–6. 10.1073/pnas.0705079104 17921253 PMC2034255

[184] Zhang DF Li J Wu H Cui Y Bi R Zhou HJ CFH variants affect structural and functional brain changes and genetic risk of Alzheimer’s disease. Neuropsychopharmacology (2016) 41(4):1034–45. 10.1038/npp.2015.232 26243271 PMC4748428

[185] Zetterberg M Landgren S Andersson ME Palmer MS Gustafson DR Skoog I Association of complement factor H Y402H gene polymorphism with Alzheimer’s disease. Am J Med Genet B Neuropsychiatr Genet (2008) 147B(6):720–6. 10.1002/ajmg.b.30668 18163432

[186] Sullivan RK Woldemussie E Pow DV. Dendritic and synaptic plasticity of neurons in the human age-related macular degeneration retina. Invest Ophthalmol Vis Sci (2007) 48(6):2782–91. 10.1167/iovs.06-1283 17525213

[187] Yoshida T Ohno-Matsui K Ichinose S Sato T Iwata N Saido TC The potential role of amyloid beta in the pathogenesis of age-related macular degeneration. J Clin Invest (2005) 115(10):2793–800. 10.1172/JCI24635 16167083 PMC1201663

[188] Patel M Chan CC. Immunopathological aspects of age-related macular degeneration. Semin Immunopathol (2008) 30(2):97–110. 10.1007/s00281-008-0112-9 18299834 PMC2441602

[189] Nozaki M Raisler BJ Sakurai E Sarma JV Barnum SR Lambris JD Drusen complement components C3a and C5a promote choroidal neovascularization. Proc Natl Acad Sci U S A (2006) 103(7):2328–33. 10.1073/pnas.0408835103 16452172 PMC1413680

[190] Heneka MT O’Banion MK. Inflammatory processes in Alzheimer’s disease. J Neuroimmunol (2007) 184(1–2):69–91. 10.1016/j.jneuroim.2006.11.017 17222916

[191] Biron KE Dickstein DL Gopaul R Jefferies WA. Amyloid triggers extensive cerebral angiogenesis causing blood brain barrier permeability and hypervascularity in Alzheimer’s disease. PLoS One (2011) 6(8):e23789. 10.1371/journal.pone.0023789 21909359 PMC3166122

[192] Chin YC Wong TY Cheung CM Cheung CY Zheng Y Mitchell P Retinal vascular caliber and age-related macular degeneration in an Indian population from Singapore. Ophthalmic Epidemiol (2014) 21(4):224–9. 10.3109/09286586.2014.926941 24945891

[193] Liew G Kaushik S Rochtchina E Tan AG Mitchell P Wang JJ. Retinal vessel signs and 10-year incident age-related maculopathy: the Blue Mountains Eye Study. Ophthalmology (2006) 113(9):1481–7. 10.1016/j.ophtha.2006.03.051 16828507

[194] Yucel YH Zhang Q Gupta N Kaufman PL Weinreb RN. Loss of neurons in magnocellular and parvocellular layers of the lateral geniculate nucleus in glaucoma. Arch Ophthalmol (2000) 118(3):378–84. 10.1001/archopht.118.3.378 10721961

[195] Yucel YH Zhang Q Weinreb RN Kaufman PL Gupta N. Effects of retinal ganglion cell loss on magno-, parvo-, koniocellular pathways in the lateral geniculate nucleus and visual cortex in glaucoma. Prog Retin Eye Res (2003) 22(4):465–81. 10.1016/S1350-9462(03)00026-0 12742392

[196] McKinnon SJ Lehman DM Kerrigan-Baumrind LA Merges CA Pease ME Kerrigan DF Caspase activation and amyloid precursor protein cleavage in rat ocular hypertension. Invest Ophthalmol Vis Sci (2002) 43(4):1077–87. 11923249

[197] Oliver JE Hattenhauer MG Herman D Hodge DO Kennedy R Fang-Yen M Blindness and glaucoma: a comparison of patients progressing to blindness from glaucoma with patients maintaining vision. Am J Ophthalmol (2002) 133(6):764–72. 10.1016/S0002-9394(02)01403-4 12036667

[198] Jindal V. Interconnection between brain and retinal neurodegenerations. Mol Neurobiol (2015) 51(3):885–92. 10.1007/s12035-014-8733-6 24826919

[199] Leske MC Heijl A Hussein M Bengtsson B Hyman L Komaroff E Factors for glaucoma progression and the effect of treatment: the early manifest glaucoma trial. Arch Ophthalmol (2003) 121(1):48–56. 10.1001/archopht.121.1.48 12523884

[200] Shields MB. Normal-tension glaucoma: is it different from primary open-angle glaucoma? Curr Opin Ophthalmol (2008) 19(2):85–8. 10.1097/ICU.0b013e3282f3919b 18301279

[201] Jain S Aref AA. Senile dementia and glaucoma: evidence for a common link. J Ophthalmic Vis Res (2015) 10(2):178–83. 10.4103/2008-322X.163766 26425322 PMC4568617

[202] Sunderland T Linker G Mirza N Putnam KT Friedman DL Kimmel LH Decreased beta-amyloid1-42 and increased tau levels in cerebrospinal fluid of patients with Alzheimer disease. JAMA (2003) 289(16):2094–103. 10.1001/jama.289.16.2094 12709467

[203] Guo L Salt TE Luong V Wood N Cheung W Maass A Targeting amyloid-beta in glaucoma treatment. Proc Natl Acad Sci U S A (2007) 104(33):13444–9. 10.1073/pnas.0703707104 17684098 PMC1940230

[204] Izzotti A Bagnis A Sacca SC The role of oxidative stress in glaucoma. Mutat Res (2006) 612(2):105–14. 10.1016/j.mrrev.2005.11.001 16413223

[205] Pinazo-Duran MD Zanon-Moreno V Gallego-Pinazo R Garcia-Medina JJ. Oxidative stress and mitochondrial failure in the pathogenesis of glaucoma neurodegeneration. Prog Brain Res (2015) 220:127–53. 10.1016/bs.pbr.2015.06.001 26497788

[206] Trovato Salinaro A Cornelius C Koverech G Koverech A Scuto M Lodato F Cellular stress response, redox status, and vitagenes in glaucoma: a systemic oxidant disorder linked to Alzheimer’s disease. Front Pharmacol (2014) 5:129. 10.3389/fphar.2014.00129 24936186 PMC4047681

[207] Hou XR Miao H Tao Y Li XX Wong IY. Expression of cytokines on the iris of patients with neovascular glaucoma. Acta Ophthalmol (2015) 93(2):e100–4. 10.1111/aos.12510 25041566

[208] Russo R Varano GP Adornetto A Nucci C Corasaniti MT Bagetta G Retinal ganglion cell death in glaucoma: exploring the role of neuroinflammation. Eur J Pharmacol (2016) S0014-2999(16):30204–7. 10.1016/j.ejphar.2016.03.064 27044433

[209] Cueva Vargas JL Osswald IK Unsain N Aurousseau MR Barker PA Bowie D Soluble tumor necrosis factor alpha promotes retinal ganglion cell death in glaucoma via calcium-permeable AMPA receptor activation. J Neurosci (2015) 35(35):12088–102. 10.1523/JNEUROSCI.1273-15.2015 26338321 PMC6605307

[210] Venkataraman ST Flanagan JG Hudson C Vascular reactivity of optic nerve head and retinal blood vessels in glaucoma – a review. Microcirculation (2010) 17(7):568–81. 10.1111/j.1549-8719.2010.00045.x 21040122

[211] Ding JD Lin J Mace BE Herrmann R Sullivan P Bowes Rickman C. Targeting age-related macular degeneration with Alzheimer’s disease based immunotherapies: anti-amyloid-beta antibody attenuates pathologies in an age-related macular degeneration mouse model. Vision Res (2008) 48(3):339–45. 10.1016/j.visres.2007.07.025 17888483 PMC2323206

[212] Hardy J Selkoe DJ. The amyloid hypothesis of Alzheimer’s disease: progress and problems on the road to therapeutics. Science (2002) 297(5580):353–6. 10.1126/science.1072994 12130773

[213] Nicoll JA Wilkinson D Holmes C Steart P Markham H Weller RO. Neuropathology of human Alzheimer disease after immunization with amyloid-beta peptide: a case report. Nat Med (2003) 9(4):448–52. 10.1038/nm840 12640446

[214] Volz C Pauly D. Antibody therapies and their challenges in the treatment of age-related macular degeneration. Eur J Pharm Biopharm (2015) 95(Pt B):158–72. 10.1016/j.ejpb.2015.02.020 25725263

[215] Andreasen N Simeoni M Ostlund H Lisjo PI Fladby T Loercher AE First administration of the Fc-attenuated anti-beta amyloid antibody GSK933776 to patients with mild Alzheimer’s disease: a randomized, placebo-controlled study. PLoS One (2015) 10(3):e0098153 10.1371/journal.pone.0098153 25789616 PMC4366075

